# Mesoporous Silica from Water-Soluble Precursors in Hybrid Polysulfone Membranes: A Strategy for Enhanced Ciprofloxacin Removal under Low-Pressure Filtration

**DOI:** 10.1021/acsomega.6c03535

**Published:** 2026-07-27

**Authors:** Diogo Augusto da Silva de Assis, Beatriz Biscola Alves, Daniel Eiras

**Affiliations:** † Postgraduate Program in Materials Engineering and Science, 28122 Federal University of Paraná, Coronel Francisco H. dos Santos Street, 210, Curitiba, Paraná 80060-000, Brazil; ‡ Department of Chemical Engineering, Federal University of Paraná, Coronel Francisco H. dos Santos Street, 210, Curitiba, Paraná 80060-000, Brazil

## Abstract

The present study introduces high-performance mixed matrix membranes (MMM) designed to provide an improved balance in the permeability-selectivity trade-off for pharmaceutical remediation. By incorporating mesoporous silica (mSiO_2_) nanoparticles, synthesized via a water-soluble precursor route, into a polysulfone (PSf) matrix, a synergistic adsorptive-filtration platform was established for ciprofloxacin (CIP) remediation. The incorporation of mSiO_2_ nanoparticles influences the mass transfer rate between solvent and nonsolvent during the membrane production process and promotes modification in the asymmetric structure of the membrane, resulting in the formation of finger-like cavities and open terminal channels with reduced wall thickness. The mSiO_2_ incorporation promoted greater and interconnected porosity, in addition to greater hydrophilicity confirmed by the surface analysis. Results demonstrate a transition from ultrafiltration-like (UF-like) to nanofiltration-like (NF-like) behavior with increasing PSf concentration. For membranes cast from the 15 wt % PSf solution the addition of mSiO_2_ increased the flux and yielded a 4-fold increase in CIP removal capacity. For the PSf-20 series membrane, the incorporation of mSiO_2_ nanoparticles enhanced water flux while maintaining high CIP rejection (83.3 ± 13.4%). Notably, the PSf-18 series emerged as the optimized formulation, achieving a superior balance between high permeability and selectivity. This performance is driven by a synergistic mechanism: while the negative surface potential facilitates Donnan exclusion of zwitterionic CIP, the internal architecture enables convective-enhanced adsorption within the mSiO_2_ micro and mesopores. These findings highlight the potential of PSf/mSiO_2_ membranes as a low-pressure (2 bar), energy-efficient platform for advanced treatment of emerging micropollutants, providing a foundational step for future applications under more complex operating conditions.

## Graphical abstract

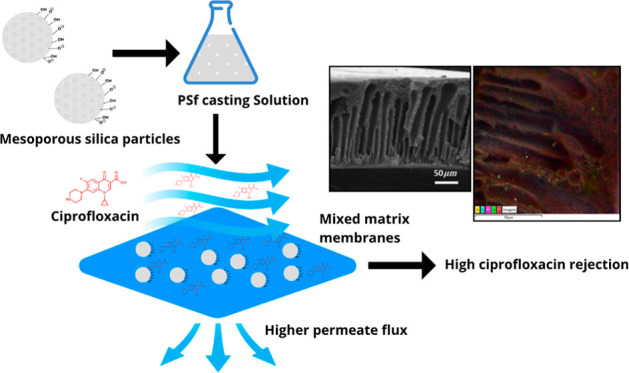

## 1 Introduction

The increasing populational growth, urbanization, and industrialization trigger a higher generation of waste, effluents, and contaminants, including pharmaceutical products, especially antibiotics.^1^ These drugs are continuously released into the environment due to their higher consumption and production. Among the numerous existing antibiotics, CIP (molecular weight (*M*_W_) = 367 Da), from the fluoroquinolone family, is one of the most used nowadays. This is mainly due to its broad spectrum of activity, being effective against Gram-negative and positive bacteria.^2^ Furthermore, it has low cost and efficient tissue penetration when compared to other antibiotics.^3^

CIP, like many other drugs, is not easily degraded, and due to the ineffectiveness of conventional municipal and industrial treatments in the complete removal of these organic compounds, it can be found in surface and groundwater.^4^ Even at low concentrations, the presence of antibiotics in aquatic environments drives the development of antibiotic-resistant pathogens and the proliferation of resistance genes, potentially threatening ecosystems and public health.^5,6^

Therefore, due to the high risk of the presence of these micropollutants in the environment, it is important that technologies capable of removing them from water bodies emerge. Several methods have been investigated to remove antibiotics and other drugs from drinking water and sewage, including ozone or electrochemical oxidation, adsorption, biological treatment, membrane separation, flocculation, phytoremediation, and photocatalysis.^7–10^ All methodologies have advantages and disadvantages, however, membrane separation technology, due to its high removal rate of low *M*_W_ organic pollutants, as well as its modularity and capacity for integration with other systems, makes it one of the most promising methods.^11–15^

Studies indicate that the membrane separation method is suitable for pharmaceutical applications due to its high efficiency.^16^ The denser pore distribution, specific surface area, and selective penetration capacity of the membrane allow the liquid to pass from one side to the other under the influence of the applied pressure. It involves pressure-driven separation through a membrane, employed by physical and chemical sieving of dissolved and undissolved pollutants. Compared to other technologies, it presents unique properties such as high resistance to chemicals, temperature, and microbiological attacks.^17,18^

Nanofiltration (NF) membranes derived from different polymers, especially PSf, have been employed in the removal of various micropollutants, including antibiotics, precisely because they allow high rejection rates (>90%) and relatively high flux when compared to RO.^19,20^ Several studies have already shown the potential for CIP and other antibiotics removal in water bodies.^11,14,16^

NF membranes, while efficient for removing pharmaceuticals, can present fouling problems, resulting from the adsorption of nonpolar solutes and hydrophobic particles or bacteria, leading to a loss of permeate flux and a reduction in membrane efficiency and lifespan.^21^ Additionally, the high transmembrane pressures typically required in NF systems contribute to elevated operational costs, limiting their broader application. Therefore, the modification of the polymeric membrane with the incorporation of hydrophilic inorganic particles, especially mesoporous structures, is a strategy to increase permeate flux values without negatively affecting antibiotic rejection and reduce operating pressures. Recent studies have demonstrated that MMM has become an alternative to overcome such challenges.^15,22–26^

The use of adsorbent structures, like silica nanoparticles, in addition to their high hydrophilicity, is a promising technology for low *M*_W_ solutes removal, such as antibiotics. Due to their high surface area, high load of active sites, and kinetics, these adsorbents are potential assets to be incorporated into NF membranes to overcome their disadvantages in water purification. Some authors have already evaluated the effect of silica nanoparticles alone or combined with other particles to simultaneously increase membrane flux and antibiotic rejection. Shakak et al. carried out the incorporation of 2 and 4 wt % of SiO_2_ in PSf matrix, promoting an increase in rejection from 66.52% of the control membrane to 79.82% and 89.81% of amoxicillin, respectively.^13^ Nikfarjam et al. synthesized a PSf membrane containing SiO_2_ and TiO_2_ nanoparticles. The authors identified an increased hydrophilicity and a higher cefotaxime rejection for the nanoparticle-containing membrane compared to the neat polymeric membrane.^27^ Sargolzaei et al. incorporated CeO_2_@SiO_2_ nanoparticles into poly­(ether sulfone) membrane matrix. The results obtained showed reduced surface roughness, enhanced membrane hydrophilicity and CIP removal.^28^ Waheed et al. fabricated a NF membrane containing functionalized mSiO_2_ nanoparticles in its active layer. The authors observed an increase in performance with the incorporation of the mesoporous structure, reaching high rejection values (>90%) for salts and sulfamethoxazole.^29^ Recently, Jillani et al. produced a NF membrane containing mesoporous triamine functionalized silica particles applied to desalination and removal of micropollutants. The results demonstrated successful incorporation of the particles into the polymeric matrix, resulting in the manufacture of membranes presenting better flux performance and selectivity for salts and antibiotic when compared to the reference membrane. It was possible to obtain a rejection of sulfamethoxazole above 96%.^30^

However, despite the advancements of these studies, a common trend is the use of dense particles and the reliance on surface functionalization to achieve high selectivity, which adds complexity and cost to the membrane fabrication process. A significant gap remains in the literature regarding the use of nonfunctionalized mSiO_2_ for antibiotic removal. While dense silica particles are limited by their low internal surface area, mesoporous structures, given their high specific surface area and abundant interaction sites, offer an expansive network of internal pores that could theoretically enhance adsorption without the need for expensive chemical grafting. Furthermore, for the synthesis of these particles, most studies employ Tetraethylorthosilicate (TEOS), a precursor that is not only costly and toxic but also requires energy-intensive, multistep protocols.^31^ Water-soluble inorganic silica precursors present a promising alternative for synthesizing low-cost mSiO_2_ particles, and the feasibility for antibiotic adsorption of these particles has been previously reported by our research group.^32^ The challenge, therefore, lies in developing a high-performance MMM that leverages the structural advantages of mSiO_2_ while maintaining a cost-effective synthesis route.

To address these limitations, this work proposes the development of a new MMM comprising mSiO_2_ nanoparticles, synthesized from an inorganic water-soluble precursor, for the modification of PSf membranes for water treatment applications. To the best of our knowledge, no previous study in the literature has evaluated MMMs incorporating nonfunctionalized mSiO_2_ derived from water-soluble inorganic precursors into PSf matrices for targeted pharmaceutical removal via low-pressure NF-like processes. The hypothesis of this work is that the incorporation of mSiO_2_ particles, with high CIP adsorption capacity, into PSf membranes will promote an increase in permeate flux while maintaining or increasing the antibiotic rejection by the composite. By synergizing the filtering capacity of the PSf matrix, the CIP adsorption potential, and the hydrophilicity of the incorporated particles, this study delivers an efficient and sustainable nanocomposite that significantly improves the traditional permeability-selectivity trade-off for pharmaceutical removal under low-pressure (2 bar) operation, as demonstrated under controlled laboratory conditions.

## 2 Materials and Methods

### 2.1 Materials

All chemical products used in this study were of reagent grade. The surfactant used for the synthesis of mesoporous particles was cetyltrimethylammonium bromide (CTAB) (98%) from Sigma-Aldrich. The silica water-soluble inorganic precursor used was from Sigma-Aldrich. Although the specific name of the precursor cannot be disclosed due to intellectual property protection, its synthesis relevance is fundamental: this precursor dissolves in the aqueous medium to release reactive inorganic silicon–oxygen species. These serve as the building blocks that undergo pH-controlled polycondensation around the structure-directing template to generate the mesoporous silica framework. CIP (98%) also from Sigma-Aldrich was used for membrane permeation tests. Sodium hydroxide (NaOH, 97%) and hydrochloric acid (HCl, 37%) were obtained commercially. For the production of the membranes, PSf Udel P-3500 LCD MB3 and *N*-methyl-2-pyrrolidone (NMP) (99%), commercially available, were used. Other solvents and reagents were obtained commercially and used as received.

### 2.2 mSiO_2_ Structure Synthesis

The mSiO_2_ particles with high CIP adsorption capacity were synthesized according to the method described by Assis et al.^32^ First, 15 g of silica inorganic precursor was dissolved in deionized water to produce a 5 wt % SiO_2_ solution (300 mL). In parallel, certain mass of CTAB was dissolved in a 2 M solution of HCl at room temperature to obtain a solution with CTAB: SiO_2_ mass ratio of 1.5/5. Then the silica solution and the homogeneous surfactant solution were mixed with a 300 mL of deionized water under stirring and at room temperature. The mixing process of these components was kept for 30 min, to guarantee complete homogeneity. Then, the resulting product was adjusted to pH = 3 and kept at rest for 8 h at room temperature. The resulting product was filtered, washed repeatedly with distilled water, and dried in an oven at 60 °C for 24 h. Finally, the synthesized sample was calcined in a muffle furnace at a heating rate of 1 °C min^–1^ and maintained for 6 h at 550 °C to remove the surfactant.

### 2.3 Characterization of mSiO_2_ Nanoparticles

The physicochemical properties, morphology, and size of the synthesized particles were evaluated using a suite of complementary techniques. First, functional groups were identified by FT-IR spectroscopy using a Bruker FTIR 70v spectrometer equipped with an attenuated total reflection (ATR) accessory. Spectra were acquired using a DLATGS detector with a KBr window in the range of 4000–400 cm^–1^ with a resolution of 4.0 cm^–1^ and 32 scans per sample. Subsequently, the structural nature and phase purity, specifically to detect salt-derived impurities, were analyzed via X-ray diffraction (XRD). These measurements were conducted on a Bruker D2 PHASER diffractometer (powder method) in the 2θ range of 5°–70°, using a step size of 0.02° and a count time of 1 s per step. Particle size distribution, pore uniformity, and morphology were examined using transmission electron microscopy (TEM) on a JEOL JEM 1200EX-II instrument. The surface area, pore volume and mean pore radius were determined from N_2_ adsorption–desorption isotherms measured at 77 K using a QUANTACHROME NOVA 2000e analyzer. Samples were degassed at 100 °C for 12 h prior to analysis, and the data were processed using standard calculation protocols. The zeta potential measurements were carried out using a Malvern Panalytical Zetasizer Nano ZS. The samples were diluted to 0.1 wt % in aqueous media, and the zeta potential variations were recorded as a function of pH (3.0, 5.0, 7.0, 9.0, and 11.0) at room temperature.

### 2.4 Hybrid Membrane Synthesis

The synthesis method of the PSf/mSiO_2_ structures membranes was based on a literature study.^33^ As described in Table 1, the casting solutions were prepared with 15 and 20 wt % of PSf. Silica nanoparticles were subsequently incorporated into the dopes at varying concentrations (0, 1, and 5 wt %), calculated relative to the dry mass of the PSf polymer. Hereafter, the composite membranes were named according to the polymer concentration in the casting solution. Thus, the PSf-15 series denotes membranes cast from a 15 wt % PSf solution, while the PSf-20 series refers to those cast from a 20 wt % PSf solution.

**Table 1:** Composition of Casting Solution

membrane name	polymer (g)	solvent (g)	mSiO_2_ (g)
M1	15	85	0.00
M2	15	85	0.15
M3	15	85	0.75
M4	20	80	0.00
M5	20	80	0.20
M6	20	80	1.00

For the formulation without the presence of adsorbent, the PSf was added to the solvent and left under agitation for 24 h until the complete mixture. The solution with incorporation of mSiO_2_ particles was prepared as follows: (i) mixing of the mSiO_2_ structures in solvent under agitation, (ii) sonication for 15 min to guarantee dispersion and complete distribution of the particles (iii) addition of the PSf and agitation during 24 h until homogeneous mixture.

After the mixing time and if no bubble formation was verified, the solutions were ready to use. Then, for the preparation of the membranes, 10 mL of each solution was deposited and spread on a glass plate to a wet thickness of 130 μm, controlled with the aid of a casting knife. After exposure of the solution on the glass plate to air for 2 s, the plate was immersed in water for 10 s at room temperature for coagulation and membrane formation. The formed membranes were then washed and stored in distilled water to remove residual solvent.^34^

### 2.5 Characterization of PSf/mSiO_2_ Nanoparticle Membranes

The samples were characterized by FTIR in order to identify chemical changes in the structure of the membranes without and with modification of the adsorbent. FTIR equipment, VERTEX 70v, Bruker equipped with attenuated total reflection (ATR) accessory was used. For ATR, the samples were placed in direct contact with the diamond crystal. All tests were carried out in the range of 4000–400 cm^–1^. The spectrum was measured with a resolution of 4.0 cm^–1^, accumulating a total of 32 scans per sample analyzed. The detector used was the DLATGS with a KBr window.

The membranes were characterized by XRD in order to evaluate changes in the structure ordering. Readings were taken on an X-ray diffractometer, D2 PHASER, Bruker. The analysis was carried out between 2θ angles of 5° and 70°, with an angular step of 0.02° and time per step of 1 s. During collection, a tube with a copper anode, 30 kV/10 mA and a diverging slit of 1 mm was used.

The rheological properties and viscosity of the polymer solutions were evaluated using a Discovery HR 10 (TA Instruments) rotational rheometer equipped with a steel conical plate geometry (40 mm diameter and 0.3 mm gap). The relationship between viscosity and shear rate was determined at a constant temperature of 25 °C and at a shear rate of 0.1 s^–1^ to 1000.0 s^–1^.

To observe the morphology and obtain information on the surface and cross-section of the membranes, they were characterized by SEM. For cross-sectional analysis, samples were fractured in liquid N_2_. The surface and cross-section of the membranes were also evaluated using energy-dispersive X-ray spectroscopy (EDS) elemental mapping in order to obtain the spatial distribution of the chemical elements. The equipment used for both SEM and EDS analyses was a FEI Quanta 450 FEG scanning electron microscope.

The zeta potential characterization of the membranes was performed using an Anton Paar SurPASS 2 electrokinetic analyzer, based on streaming potential measurements. The analysis was conducted in a pH range of 3–10, with the pH automatically adjusted by titrating the electrolyte solution with 0.05 M HCl. Zeta potential values were calculated from an average of four measurements at each pH point.

The porosity of the membranes was determined by the gravimetric method, which involves, first, a step of complete drying of a previously weighed membrane sample, and then the submersion of the sample in distilled water for 24 h to ensure saturation.^35^

After this period, the membrane was removed from the water and weighed again. The porosity of the membrane was calculated using eq 1

1 $$porosity\;\left(\%\right)=\left(\frac{\frac{\left(\omega_{w-}\omega_{d}\right)}{\rho_{k}}}{\frac{\omega_{w-}\omega_{d}}{\rho_{k}}+\frac{\omega_{d}}{\rho_{p}}}\right)$$

where ω_w_ and ω_d_ represent the weight of the membrane after immersion in water and after drying, respectively. The symbols ρ_k_ and ρ_p_ represent the density of water (0.998 g cm^–3^) and PSf (1.24 g cm^–3^), respectively.

The average pore radius (*r*_m_) was calculated according to the Guerout–Elford–Ferry equation (eq 2)^36^

2 $$r_{m}=\sqrt{\frac{\left(2.9-1.75\varepsilon \right)8\eta lQ}{\varepsilon A\Delta P}}$$

where *r*_m_ denotes the average pore radius of the membrane (m); η represents the viscosity of water (8.9 × 10^–4^ Pa s); l denotes the membrane thickness (m); Q the volume of water permeated per unit time (m^3^ s^–1^); and Δ*P* the operating pressure (Pa).

In order to determine the hydrophilic character of the membranes, the contact angle test was performed by the sessile drop method and hydrophilicity with the contact angle test. In this test, water droplets were deposited onto the membrane surface using a microsyringe. Images were captured for each sample, and the contact angle was measured via digital software. A lower contact angle indicates higher membrane hydrophilicity.

### 2.6 Permeation and Rejection Test

The membranes produced were evaluated in relation to water permeability and CIP rejection potential, using the experimental unit as illustrated in Figure 1.

**Figure 1:**
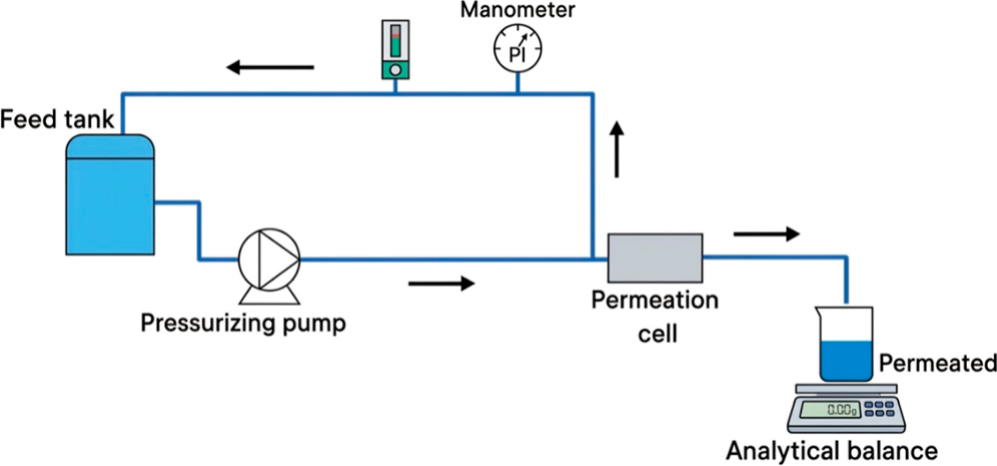
Scheme of the experimental unit used for membrane permeability and performance tests.

The membranes were tested in triplicate using a filtration process with DI water and an aqueous CIP solution at a concentration of 12.5 ± 2 mg L^–1^. All experiments were conducted at room temperature (25 ± 2 °C) in a crossflow system. For each membrane filtration process, a compaction step was first performed for 1 h at a pressure of 6.9 bar. During this step, the membrane must be subjected to a higher pressure than the operating pressure to prevent further compaction during the testing phase. Subsequently, the test continued at pressures of 2, 4, and 6 bar for 1 h at each value. Therefore, the filtration process for each sample lasted a total of 4 h. At intervals of 5 min, the permeate mass was recorded using an analytical balance to calculate the flux for each sample.

For the quantification of CIP, samples were collected from the feed tank at the beginning and end of the filtration process. Additionally, at the end of each 1 h step, a sample of the permeate was collected to identify the flux and rejection for each tested pressure. The resulting CIP concentration was determined at 277 nm using ultraviolet–visible (UV–vis) spectroscopy. The flux of each synthesized membrane was calculated using eq 3

3 $$J=\left(\frac{L}{\Delta t\times A}\right)$$

where *J* represents the flux in L m^–2^ h^–1^; *A* is the surface area of the membrane (*m* ^2^) and Δ*t* is the permeation time (h). To describe the behavior of the membrane separation, the rejection was determined according to eq 4:^37^

4 $$R_{m}=\left(1-\frac{C_{P}}{C_{A}}\right)\times 100$$

where *R*_m_ is the membrane rejection in %, *C*_A_ is the concentration of CIP in the feed, and *C*_p_ refers to the concentration of CIP in the permeate.

## 3 Results and Discussion

### 3.1 Characterization of mSiO_2_ Nanoparticles

The XRD characterization analysis was carried out to investigate the structural characteristics and degree of crystallinity of the synthesized materials. As shown in Figure 2a, the amorphous halo identified at 2θ = 15–30° is related to the diffraction behavior of the amorphous structure of SiO_2_ (JCPDS 29-0085). The FTIR absorption spectra of mSiO_2_ sample is presented in Figure 2b. Absorption bands located at approximately 430 cm^–1^ and 800 cm^–1^ are associated with the vibrational modes of Si–O–Si bonds. In addition, the bands observed in the 1000–1150 cm^–1^ region correspond to the characteristic stretching vibrations of Si–O bonds. No absorption bands were detected near 1500, 2855, or 2916 cm^–1^, which are typically assigned to the N–H and C–H stretching vibrations of CTAB, indicating the effective removal of the surfactant.^38^ The TEM images are presented in Figure 2c. As expected, the nanoparticles exhibit sizes below 200 nm, and a well-ordered hexagonal mesoporous arrangement can be observed among the particles. The pore size was estimated to be in the range of 2–5 nm. According to the IUPAC classification, materials with pore diameters between 2 and 50 nm are classified as mesoporous.^39^ Based on the zeta potential analysis as a function of pH, as shown in Figure 2d, the mSiO_2_ exhibits a predominantly electronegative surface across almost the entire pH range evaluated (pH 3 to 11), varying from approximately −12 mV to −28 mV. This behavior is driven by the progressive deprotonation of surface silanol groups (Si–OH), where the release of H^+^ ions at higher pH values converts these groups into negatively charged siloxane species (SiO^–^). The curve demonstrates a sharper drop in surface charge between pH 3 and 5, tending toward a plateau of stabilization at more alkaline values, which reflects the strong hydrophilic affinity and electrostatic interaction potential of the nanoparticle with cationic solutes at neutral and basic pH.^32^ As shown in Figure 2e the N_2_ sorption analysis revealed a composite Type I/IV isotherm, evidencing a highly developed hierarchical micromesoporous structure. Most notably, the material exhibited an elevated Multi-Point BET specific surface area of 664 m^2^ g^–1^ and the DFT cumulative surface area reached 1145 m^2^ g^–1^ indicating a significant contribution from micropores that are typically underestimated by classical models. Furthermore, the structural duality of the particles was confirmed by a BJH desorption pore volume of 0.379 cm^3^ g^–1^, alongside modal radius of 18.09 Å and 1.75 Å calculated via the BJH and DFT methods, respectively.^40^

**Figure 2:**
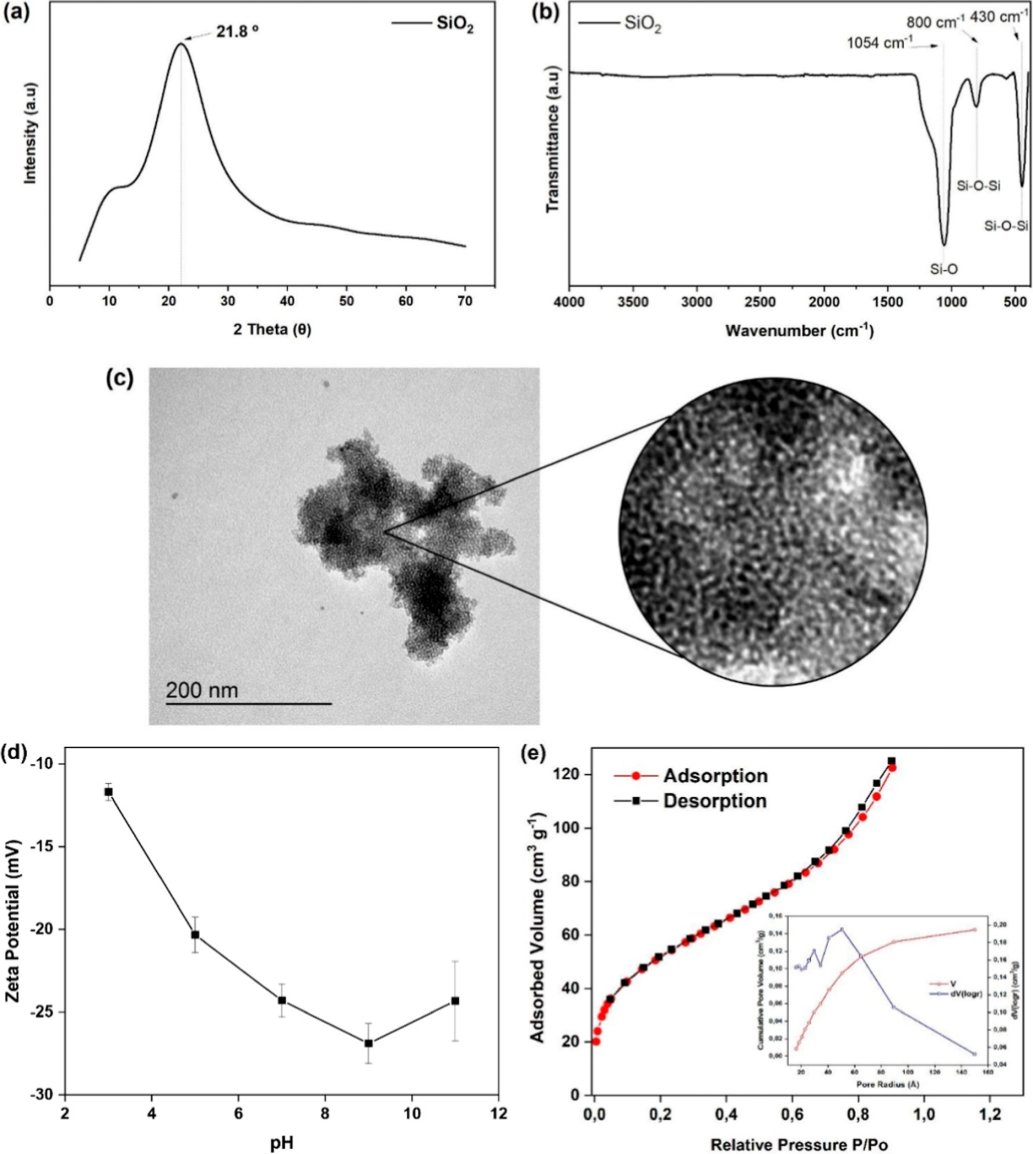
(a) XRD pattern (b) FTIR spectrum (c) TEM image (d) zeta potential and (e) surface area and pore volume analysis for the synthesized mSiO_2_ particles.

### 3.2 Characterization of Hybrid PSf/mSiO_2_ Nanoparticle Membranes

#### 3.2.1 Rheological Behavior

The viscosity of the polymer solution plays a crucial role in the formation and final properties of the membrane. Adjusting the viscosity can influence the demixing rate and gelation dynamics.^41^ During the phase inversion process, higher viscosity reduces the exchange rate between the solvent and nonsolvent, leading to the formation of denser membranes with smaller pore sizes. The thickness of the membrane is also a property strongly influenced by this exchange rate, with less viscous solutions tending to form membranes with thinner layers, influencing their permeability rate.^42^

Figure 3 illustrates the data obtained for the viscosity test. It was shown, as expected, that the viscosity values obtained for solutions with 15 wt % PSf are lower than those with higher polymer concentration. This is explained by the greater presence of PSf chains in relation to the solvent, leading to increased interactions between chains, such as entanglement and intermolecular bonds, which increase resistance to flow.^43^ It was possible to observe a sharp drop in viscosity with an increase in the shear rate, until a value was reached, after which no further variations occurred. This phenomenon indicates that all analyzed samples showed non-Newtonian fluid behavior.^41^

**Figure 3:**
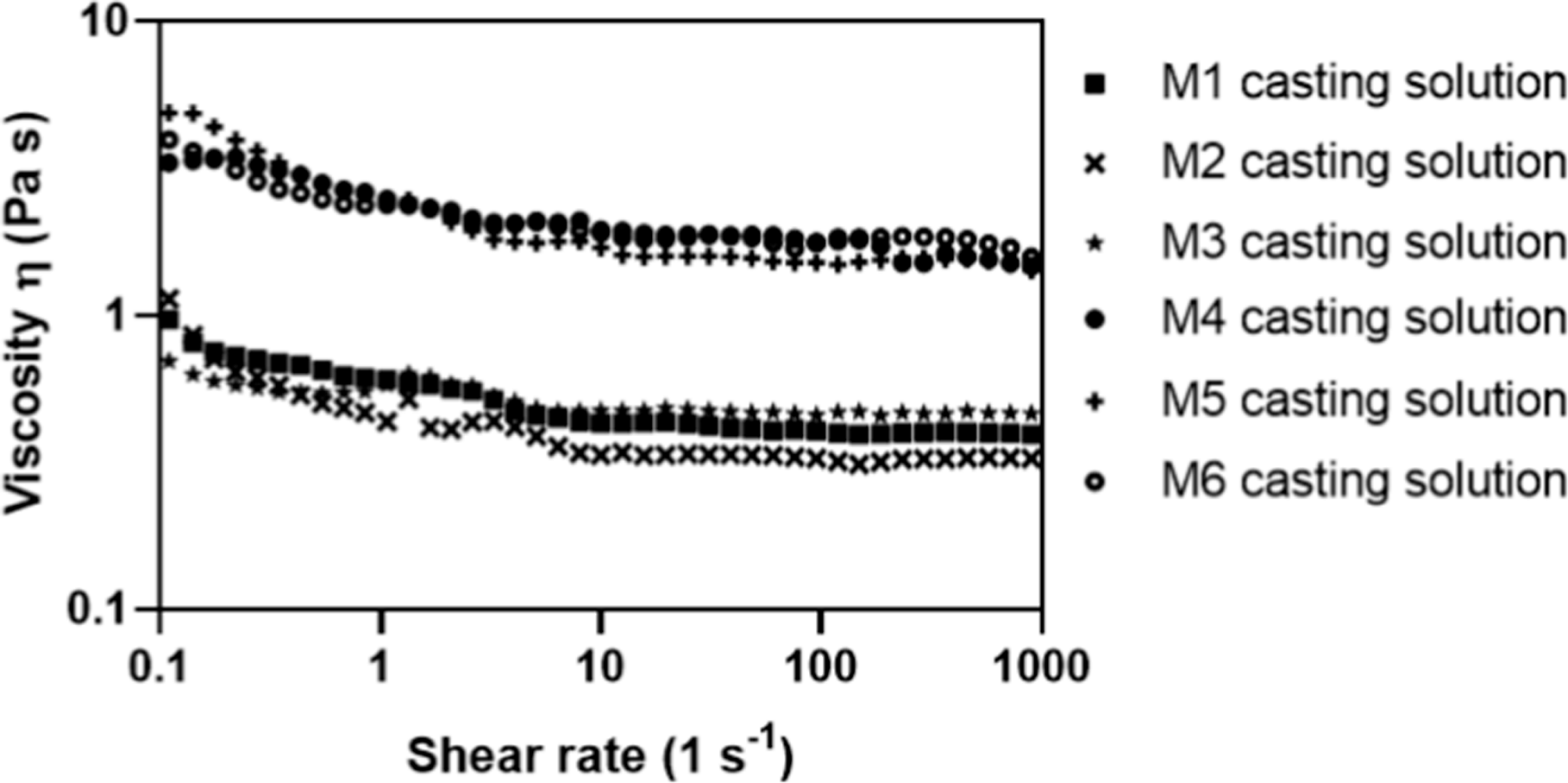
Viscosity as a function of shear rate for different synthesized membranes.

Furthermore, for the 15 and 20 wt % PSf dope solutions, it was observed that the incorporation of mSiO_2_ nanoparticles, for lower shear rates, promotes an increase in viscosity. For the less concentrated polymer solution, however, for higher shear rate values, the viscosity does not present substantial variation. Therefore, it is an indication that the presence of particles, under the conditions analyzed, increases the solution’s resistance to starting the flow.^44,45^

The 20 wt % PSf dope solutions behave differently at higher shear rates, in which the viscosity of the M6 casting solution sample presents higher viscosity than the other membranes. This probably arises from the greater interaction at higher concentration regimes, increasing the contact zones between the particles and the polymer at both lower and higher shear energies.

#### 3.2.2 Structural and Chemical Characterization (XRD, FTIR and EDS Analysis)

The XRD diffraction patterns of the synthesized membranes are shown in Figure 4a in both polymeric concentrations, it is possible to identify the diffraction pattern of PSf, represented by a large halo corresponding to its amorphous structure, at approximately 2θ = 17.6°.

**Figure 4:**
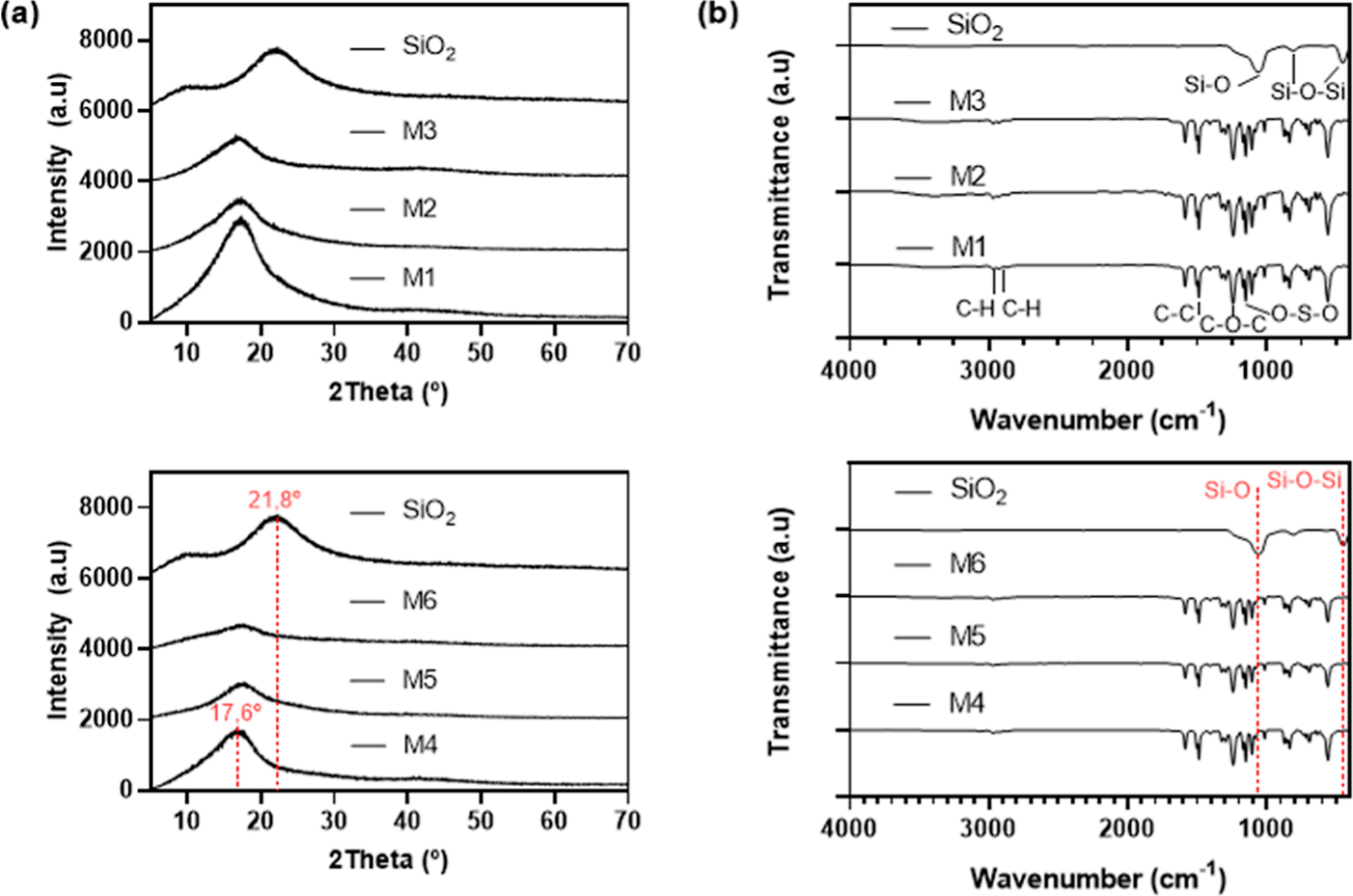
(a) XRD diffractograms for M1–M6 membranes (b) FTIR spectrum for M1–M6 membranes.

Regarding PSf/mSiO_2_, as detailed in Table 2, it is observed that the increase in the concentration of SiO_2_ nanoparticles tends to reduce the intensity of the PSf halo, with a slight shift to a higher angle and a broadening of the halo. In the PSf-15 series, the diffraction angle shifted from 17.61 to 17.85, whereas in the PSf-20 series, the shift was more pronounced, reaching 18.35 in sample M6. This increase in the diffraction angle reflects a direct reduction in the interchain spacing (*d*-spacing), which decreased from 5.05 Å to 4.83 Å. This suggests a structural reorganization of the polymeric matrix induced by the presence of mSiO_2_ particles, restricting the polymer chains mobility and promoting denser molecular packing. Simultaneously, the drastic drop in halo intensity, particularly evident in the M6 membrane, signals a disruption in the polymer’s short-range order. The silica acts as a structure that hinders the statistical alignment of the PSf chains, resulting in increased amorphous disorder.^46–48^

**Table 2:** XRD Parameters

samples	2θ (°)	*d*-spacing (Ȧ)
M1	17.61	5.03
M2	17.75	4.99
M3	17.85	4.96
M4	17.55	5.05
M5	17.95	4.94
M6	18.35	4.83

Figure 4b shows the absorption spectra obtained for the synthesized membranes. In addition, the absorption spectrum of the calcined particle is also presented for comparison with the other spectra. Regarding the PSf membrane, for both concentrations’ series, it is possible to observe the standard absorption behavior of the polymer. Based on the literature it was possible to identify for the wavenumber of 1151 cm^–1^ the O–S–O stretching band, for 1244 cm^–1^ the C–O–C stretching, and two substantial absorptions at 1487 cm^–1^ and 1585 cm^–1^ that are related to the aromatic C=C vibrational bonding in the PSf group.^46,49^ In addition, it was also possible to identify absorption bands at 1020 cm^–1^ and 830 cm^–1^, which are related to the C–H stretching of the aromatic ring of the PSf.^50^ The incorporation of mSiO_2_ nanoparticles caused no substantial alterations or new distinct bands in the FTIR spectra of the composite membranes compared to pristine PSf membrane. This behavior suggests that the nanoparticles are covered by the polymer matrix within the top layer rather than heavily exposed at the absolute surface, limiting their detection by the surface-sensitive FTIR analysis.^13^

To evaluate the elemental chemical composition and spatial distribution of the mSiO_2_ nanoparticles within the polymeric matrices, cross-sectional elemental mapping was performed for M4 and M6 membranes (Figure 5). As expected, the control membrane exhibited high, uniform concentrations of Carbon (C), Oxygen (O), and Sulfur (S), which are characteristic elements of the PSf backbone, showing no traces of SiO_2_ particles. In contrast, the modified membrane displayed an additional Silicon (Si) signal confirming the successful incorporation of the mSiO_2_ particles within the functionalized structure.^51^ The cross-sectional elemental mapping gives a clear picture of how the nanoparticles are distributed within the PSf matrix. While the C and S signals map out the polymer structure and its finger-like macrovoids, the Si and O maps actually show two things happening at once. First, there is a steady background signal showing that a good portion of the silica is well-dispersed throughout the membrane. At the same time, we can see several high-intensity zones, which point to the formation of significant particle agglomerates. This coexistence shows that while part of the nanomaterial disperses well, the high filler loading enhances particle–particle interactions, leading to these localized clusters.

**Figure 5:**
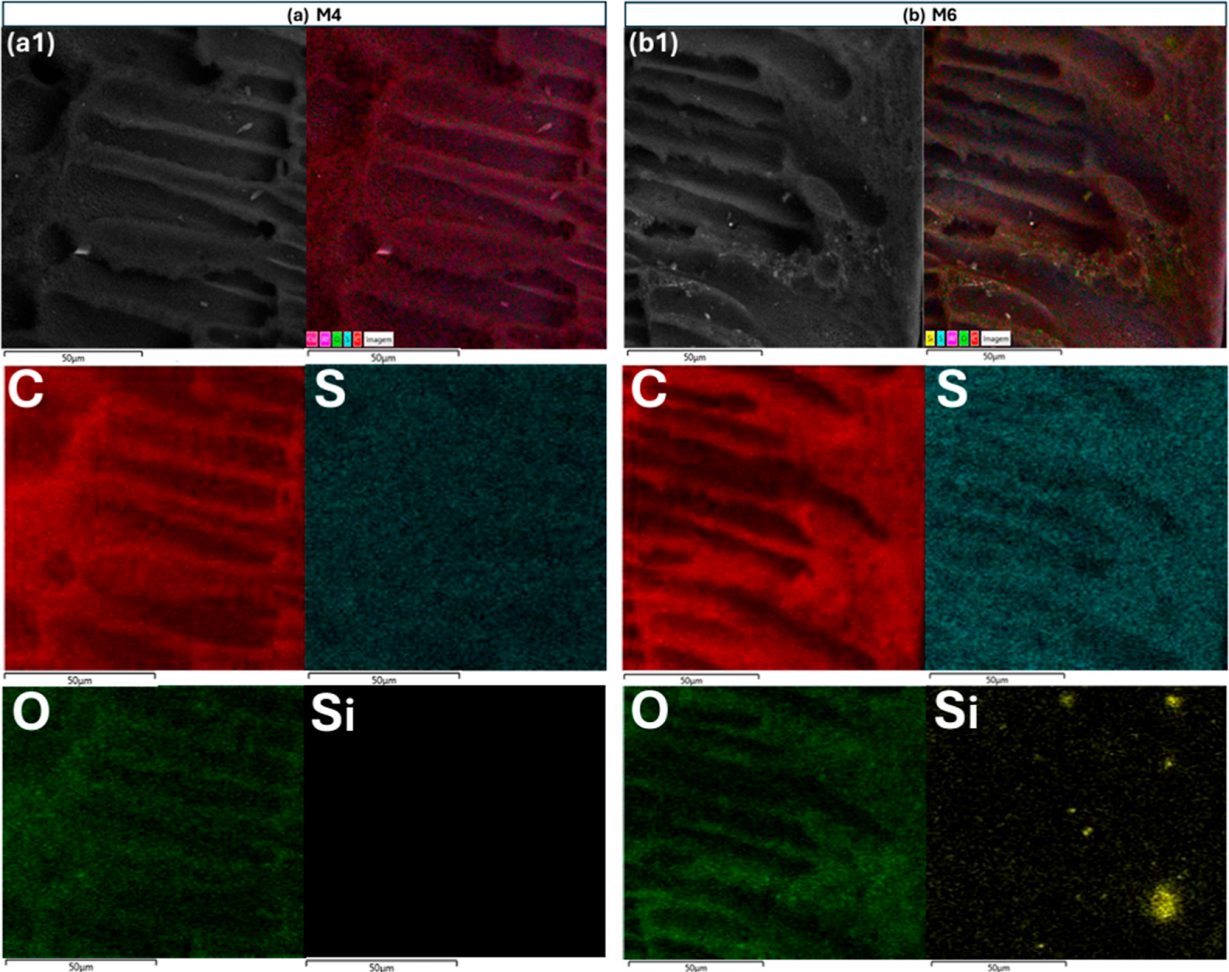
Cross section SEM-EDS map of (a) M4 and (b) M6 membranes. SEM images (a1,b1) and SEM-EDS maps of oxygen (O), sulfur (S), carbon (C) and silicon (Si).

#### 3.2.3 Morphology and Porosity

The morphological structure of the surface and cross-section of the PSf-15 series membranes were analyzed by the FESEM technique (Figure 6). From the images of the samples analyzed surface, it was possible to identify that M1 presents a denser surface structure when compared to the other membranes. With the increase in the concentration of SiO_2_, the formation of defects (pin holes) and greater roughness on the surface was noted. This possibly occurred due to the breakdown of the homogeneity of the polymeric chains on the surface caused by the presence of the SiO_2_ nanoparticles, a phenomenon observed by XRD. The increase in roughness may also occur due to the accumulation of nanoparticles, which have already been identified by other studies.^52,53^

**Figure 6:**
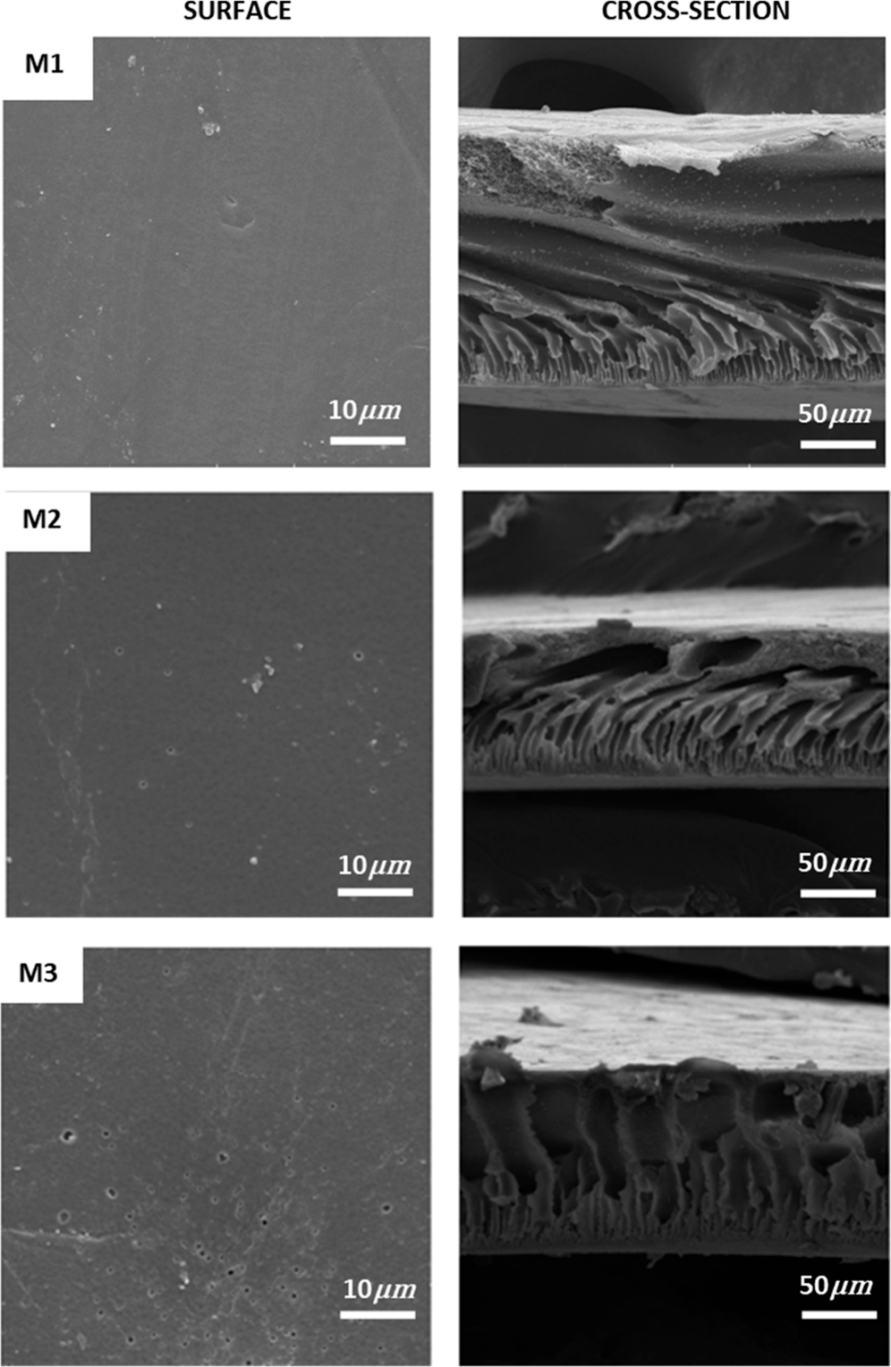
Surface and cross-section FESEM images for M1, M2 and M3 membranes.

Regarding the cross-section of the membranes, it was observed that all synthesized membranes have an asymmetric structure, formed by three layers: a superior thin and less porous layer, a porous sublayer, and a small sponge-like portion in the bottom layer. As observed in the cross-sectional images, the M1 membrane exhibits a thicker surface layer and a small number of finger-like channels, with most being inclined and undefined. These observations are consistent with other studies that have characterized PSf membranes.^35,54–57^

As can be seen in Figure 6, the incorporation of mSiO_2_ promotes a modification in the asymmetric structure of the membrane, so that the thickness of the surface layer decreases, and the porous structure of the membrane changes from a sponge-like state to finger-like cavities and open terminal channels with a thinner thickness. The reduction in the dense layer results in an increase in porosity and, therefore, the flux of the membrane.^13,58^

For the pristine PSf membrane, it can be observed that the macrovoids and finger-like pores were separated by the spongy structure, and a clear boundary can be observed between the sublayer and the bottom layer. However, the macrovoids and finger-like pores were almost merged for M3, reducing the boundary between the sublayer and the bottom layer. With the increase in mSiO_2_ content, the finger-like macrovoids increased along with the membrane thickness and became wider near the posterior part of the membrane.^59^

According to the literature the method of preparing polymeric membranes by phase inversion, in particular the exchange rate between solvent and nonsolvent, directly influences the morphology and substructure of the membrane.^26,34^ Depending on the composition of the polymer solution and the exchange rate, which can be instantaneous or delayed, different types of porous structure are formed. At faster exchange rates, finger-shaped structures are obtained, on the other hand, systems with slower phase separation led to the formation of a porous sponge-shaped substructure. Therefore, the change in morphology identified in SEM images is due to the increase in the displacement speed of the solvent and nonsolvent due to the presence and hydrophilic nature of mSiO_2_ nanoparticles. The particles act as nucleation sites due to their hydrophilic nature, resulting in the acceleration of the phase separation rate, and consequently increase the nuclei in the internal layers, resulting in more interconnected macrovoids.^60,61^ Furthermore, due to the presence of mSiO_2_ nanoparticles, phase separation in the middle regions occurs more homogeneously, generating the suppression of the dense middle layer.^34^

In addition, the incorporation of nanoparticles promotes a structural disruption of the polymeric chains, as evidenced by the reduction in XRD halo intensity. While the decreased *d*-spacing suggests localized chain tightening, the overall fractional free volume may increase due to the creation of interfacial voids, which also explains the higher porosity observed.^62^

Figure 7 illustrates the morphology of the surface and cross-section of PSf-20 series membranes. Similar to what was observed for the surface of the PSf-15 series membranes, the incorporation of 1 wt % mSiO_2_ nanoparticles promotes greater roughness and porosity on the surface of the polymeric membrane. However, in this case, the increase to 5 wt % addition of particles made the surface denser, and in addition to not observing visible pin holes, the presence of agglomerated particles was identified.^63^ In addition to agglomerated particles, for a higher level of adsorbent incorporation, surface defects were also observed, such as particle drag and lower homogeneity. This can be derived from the membrane formation process, using the phase inversion method. This method, depending on the characteristics of the polymeric solution, can lead to the formation of defects in the membrane, such as cavities or voids, variations in the roughness of the membrane surface and segregation or migration of active ingredients, leading to a nonuniform distribution of these components, affecting performance of the composite.^56,64^

**Figure 7:**
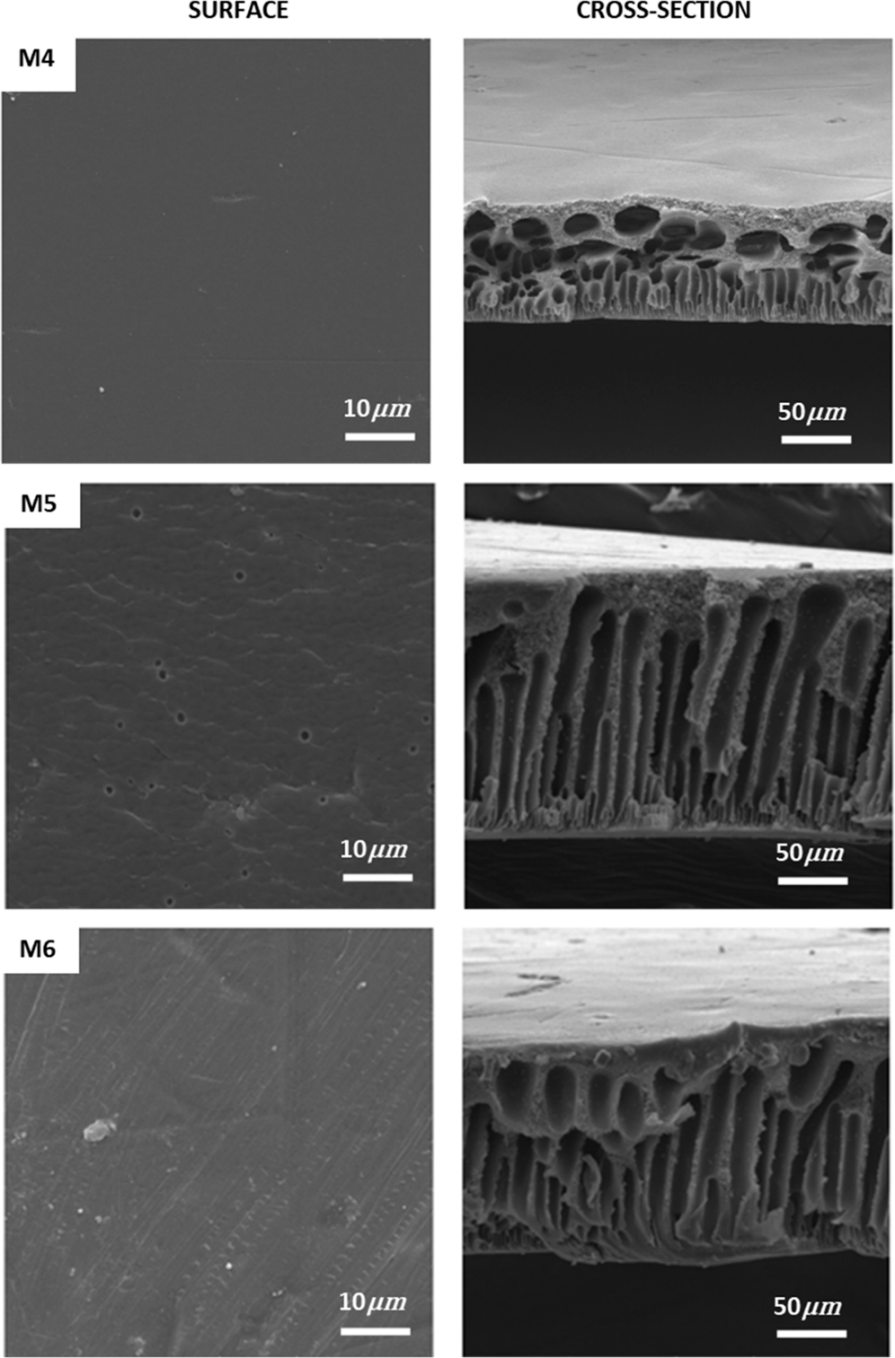
Surface and cross-section FESEM images for M4, M5 and M6 membranes.

The cross-sectional analysis showed that for all the synthesized membranes, it was possible to observe an asymmetric structure, formed by three layers. The images reveal that M4 has a thicker surface layer, small finger-like channels, and drop-shaped macrovoids. The M5 has wider and more interconnected finger-like channels, similar to what was observed for the PSf-15 series membranes. However, for the membrane with a higher incorporation of mSiO_2_, less definition of the channels and interconnectivity between the pores was observed.

This behavior has been evidenced in other studies when excess of SiO_2_ is incorporated into the polymeric solution.^50,55,60^ In general, the porosity characteristics of the membrane depend on the mass transfer rate of the polymeric solution during the phase inversion process.^13^ With the increase in the SiO_2_ content in the polymeric solution, its viscosity tends to increase, and the rate of exchange of solvent and nonsolvent in the phase inversion process is retarded. In addition, pore blockage can occur due to the high concentration of particles.^61,65^ As a result, a less porous membrane structure and a denser surface are formed.

The agglomeration of particles results from the intrinsic attraction between them, combined with their high surface area and the hydrophobic nature of the polymeric matrix. This agglomeration results in voids formed at the polymer-mSiO_2_ cluster interface due to poor interfacial adhesion between the mSiO_2_ nanoparticles and the PSf matrix. This behavior is consistent with the results obtained by EDS and XRD, related to the increased disorder of the structure with the incorporation of mSiO_2_.^54^

Membrane porosity can be defined as the weight of pure water retained in 1 m^3^ of the membrane structure. It is an important parameter in membrane separation that affects the performance and mechanical resistance of the membrane. The incorporation of nanoparticles into membranes can influence porosity in several ways, depending on their type, concentration and dispersion, as well as the conditions of the membrane manufacturing process. Figure 8a,b illustrate the porosity data for the synthesized membranes.

**Figure 8:**
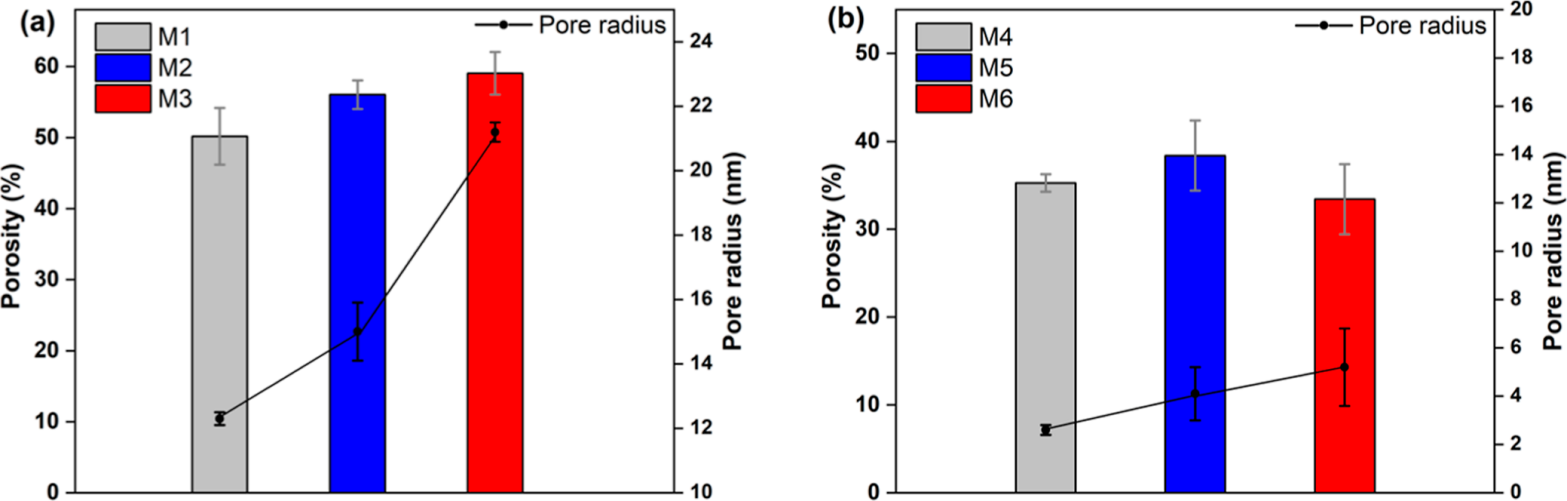
Porosity and Pore radii values of synthesized membranes: (a) PSf-15 series and (b) PSf-20 series.

The results for the PSF-15 series membranes demonstrated that increasing the mSiO_2_ additive content led to a corresponding increase in porosity. This trend agrees with both the morphological observations from the SEM analysis and the enhanced hydrophilicity revealed by the water contact angle measurements.

According to the literature, the incorporation of nanoparticles can lead to the formation of additional pores, due to the creation of tension points between the polymer and the inorganic material, during the membrane synthesis process.^50,66^ Furthermore, as seen in the SEM analysis, the increase in pore size and the greater interconnectivity between them can lead to a greater volume of water entry and, therefore, greater porosity.

The PSf-20 series membranes, as expected, showed lower porosity, due to the higher polymer concentration and formation of a denser structure. However, a similar trend driven by increasing the adsorbent loading was not observed for these matrices, given that the obtained porosity results remain within a highly comparable nominal range. Based on the average porosity results, an increasing trend was observed, but a high variation was observed. This may be related to defects generated by the high concentration of nanoparticles and viscosity of the polymer solution, resulting in poor dispersion of the particles and the development of a heterogeneous membrane structure.^67^

Furthermore, another reason that may explain the standard deviation obtained from the results is that the assessment of porosity using the gravimetric method may not be the most appropriate for membranes with a high concentration of polymer. This is because PSf is a hydrophobic polymer and, therefore, the higher its concentration in the polymeric solution, the more hydrophobic the resulting membrane tends to be. As the gravimetric method evaluates the amount of water that fills the membrane pores, it is assumed that the entire empty region has been completely occupied. If this has not occurred, in specific regions of the membrane, discrepant results may occur, increasing the deviation of the analysis.^54^

Figure 8 also presents the pore radii values obtained for the synthesized membranes. Based on the results, it is possible to observe that for the PSf-15 series membranes, the incorporation of 1 and 5 wt % of adsorbents resulted in a pore radii increase of approximately 22% and 72%, respectively. Analyzing the values obtained for the PSf-20 series membranes, smaller pore radii are identified, as expected. The incorporation of 1 wt % of mSiO_2_ resulted in an increase in pore size by approximately 59%. However, compared to what was seen for the PSf-15 series membranes, the addition of a greater quantity of particles did not result in a substantial difference when comparing the M5 and M6 membranes.

#### 3.2.4 Surface Performance (Contact Angle and Zeta Potential)

Membrane hydrophilicity is a critical factor for enhancing pure water flux and mitigating fouling. Fouling on the membrane surface can be effectively suppressed by reducing its surface hydrophobicity. In this context, contact angle measurement is the most widely adopted method to evaluate surface wettability and characterize membrane hydrophilicity.

Figure 9 illustrates the results obtained by the sessile drop contact angle test for different contact times and different synthesized membranes. According to Figure 9a, it is possible to identify that M1 presents a contact angle above 90° at *t* = 0 and reaches a value of 88.67° after 60 s. The M2 membrane already showed a lower angle value, approximately 82.67° at *t* = 0° and 80.70° at *t* = 60 s. That is, the incorporation of only 1 wt % of SiO_2_ nanoparticles has already made the membrane more hydrophilic, reducing the angle formed by the contact with the water drop by about 9%. With the incorporation of 5% of hydrophilic nanoparticles, it was possible to observe an even greater reduction in the contact angle value, being 69.33° for *t* = 0 and 67.80 for *t* = 60 s.

**Figure 9:**
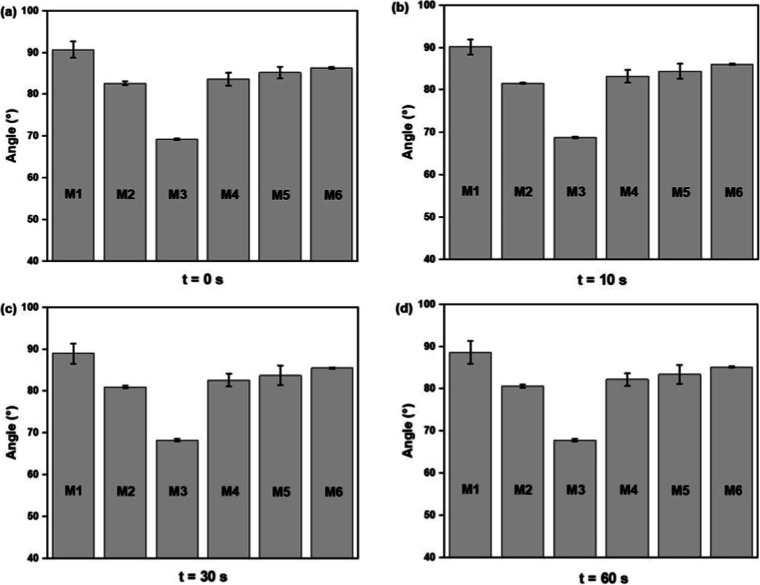
Dynamic contact angle analysis of the synthesized membranes: (a) *t* = 0 s, (b) *t* = 10 s, (c) *t* = 30 s and (d) *t* = 60 s.

A similar trend was found in previous studies that showed that the hydrophilicity of membranes increased after the addition of SiO_2_ in the membrane formulation.^50,59–61^ This agrees with the morphology results observed by SEM and porosity tests for PSf-15 series membranes. The greater presence and size of pores on the surface directly influences the contact angle formed between the water and the polymeric layer. More porous membranes tend to have a larger effective surface area, influencing the spreading of the water droplet.^66^ Additionally, the presence of accessible hydroxyl groups within the pore structure leads to an increase in the membrane’s hydrophilicity, synergistically assisting the overall decrease in the contact angle.^13,53^ Therefore, the results showed that the M3 presented the greatest reduction in the contact angle and, therefore, is the most hydrophilic membrane among the synthesized ones.

However, as illustrated in Figure 9b, the PSf-20 series membranes did not show the same behavior as the PSf-15 series. This can be explained by the comparable nominal range of the membrane porosity, combined with the nonoptimized nanoparticle dispersion of mSiO_2_ particles in the membranes after the phase inversion process. In addition to PSf being an intrinsically hydrophobic polymer, the 20 wt % PSf polymer solution has a higher viscosity when compared to the 15 wt % solution, which may hinder the complete dispersion of the mSiO_2_ particles. Thus, the lower overall porosity, agglomeration effect, and the limited access to the hydroxyl groups on the surface results in membranes that maintain a wetting behavior and hydrophilicity similar to the pristine reference membrane.^34,46^

Furthermore, based on the results obtained by SEM, it was possible to observe defects and greater surface roughness. According to the literature, surface roughness significantly affects the contact angle between water and the polymeric selective layer.^53^ Based on the Wenzel model, rougher surfaces with an inherently hydrophobic character tend to exhibit larger water contact angles due to the wetting behavior of the water droplet on the textured matrix.^20,64^ Additionally, this roughness induces surface heterogeneity, creating alternating hydrophilic and hydrophobic domains that lead to an uneven distribution of water molecules and subsequent variations in the measured contact angle.

The zeta potential of the membranes is shown in Figure 10. The electrokinetic behavior of PSf membranes shows a strong pH dependence, characterized by a decrease in zeta potential values as the solution pH increases.^65^ Although the chemical structure of PSf lacks intrinsic ionizable groups in its main chain, the membrane surface acquires an electrical charge through specific adsorption mechanisms at the polymer–solution interface. At pH values > 4, the surface charge becomes predominantly negative, probably due to the preferential adsorption of anions on the hydrophobic surface, while cations tend to be repelled or less adsorbed.^66^

**Figure 10:**
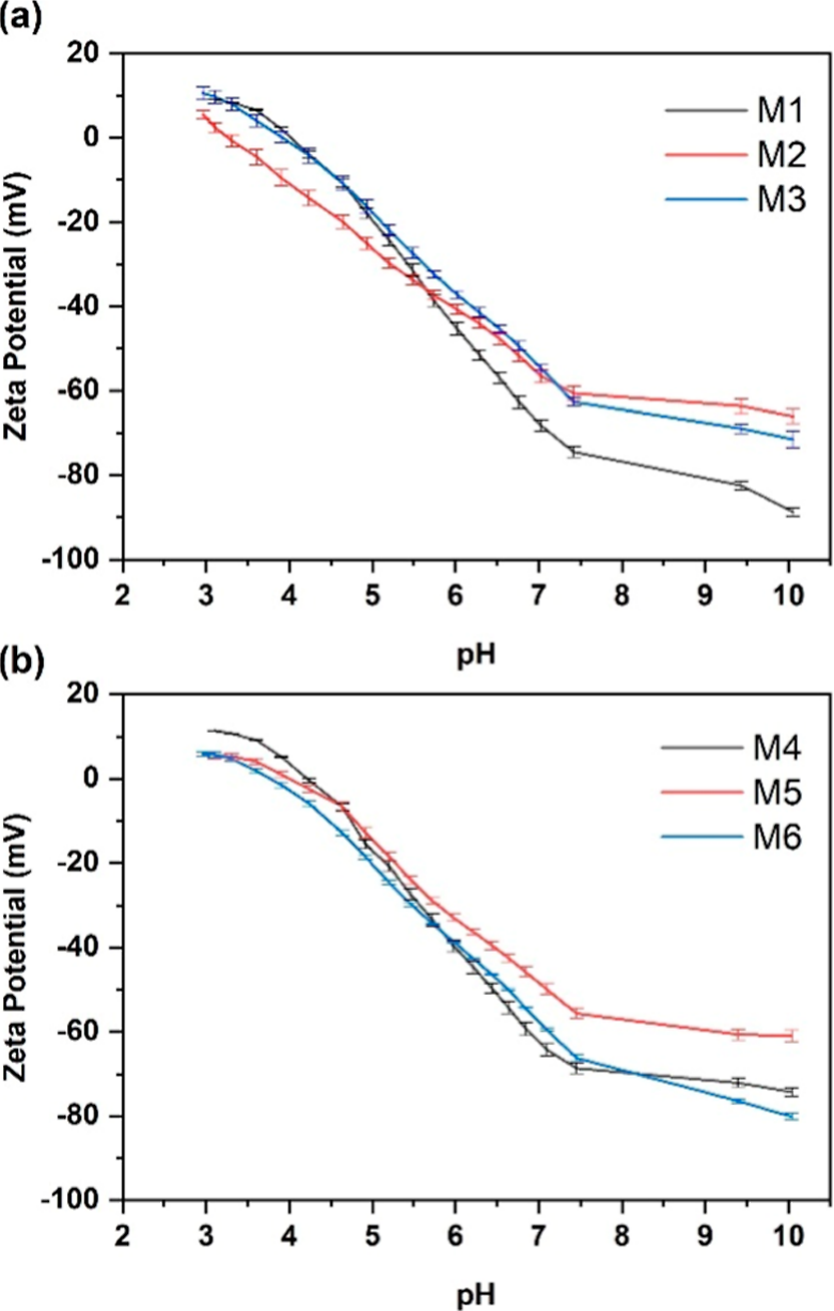
(a) Zeta Potential behavior at different pH values for M1, M2 and M3 membranes and (b) M4, M5 and M6 membranes.

It was possible to identify that the incorporation of mSiO_2_, despite its negative character, did not increase the negative charge of the PSf membrane. This lack of significant alteration in surface charge suggests a partial polymer coverage of the nanoparticles within the top layer, preventing their negative charges from substantially influencing the overall surface potential. Alternatively, it indicates that the density of particles exposed at the top interface was insufficient to overcome the intrinsic charge density of the PSf matrix itself.^52^

The increase in roughness, resulting from the presence of nanoparticles on the surface, may also be a determining factor as it may lead to an underestimation of the absolute value of the zeta potential. The standard Helmholtz-Smoluchowski equation used by the SurPASS equipment assumes a perfectly smooth surface. When the surface is rough, the shear plane and the flow lines of the electrolyte are altered, leading to an underestimated zeta potential reading.^67^

#### 3.2.5 Separation Performance (Permeability and Rejection)

Tables 3 and 4 present the flux results obtained by passing water and CIP solution through PSf-15 series membranes. Based on these results, and considering the operating pressures and estimated pore radii, these synthesized matrices exhibit characteristic ultrafiltration-like (UF-like) separation behavior. As expected, the pure water flux increased linearly with increasing pressure from 2 to 6 bar, indicating that membrane compaction had been successfully completed prior to the measurements.

**Table 3:** Pure Water Flux Results for Membranes with 15 wt % PSf and Different Adsorbent Contents

	pure water flux (L m^–2^ h^–1^)		
membrane code	2 bar	4 bar	6 bar
M1	29.4 ± 0.9	55.4 ± 1.4	70.4 ± 2.7
M2	55.9 ± 1.4	83.3 ± 33.9	126.7 ± 45.1
M3	110.9 ± 2.7	143.9 ± 24.8	180.6 ± 26.2

**Table 4:** CIP Solution Flux Results for Membranes with 15 wt % PSf and Different Adsorbent Contents

	12.5 mg L^–1^ CIP solution flux (L m^–2^ h^–1^)		
membrane code	2 bar	4 bar	6 bar
M1	26.3 ± 7.6	63.3 ± 3.0	68.9 ± 26.2
M2	71.5 ± 13.1	105.4 ± 36.3	111.4 ± 29.2
M3	78.6 ± 43.6	104.9 ± 31.0	142.5 ± 31.9

The incorporation of 1 and 5 wt % of mSiO_2_ promoted an increase in the flux of water and CIP solution, especially at a pressure of 2 bar. This is in accordance with the characterization tests that identified an increase in porosity and pore size, caused by the presence of these particles, in addition to an increase in the hydrophilic character of the membrane. As evidenced by the results obtained in this work and in other studies in the literature, the incorporation of mSiO_2_ can result in increased porosity due to the formation of macrovoids that enhance pore interconnectivity and size.^45,68^ Furthermore, the presence of such particles can lead to the formation of a less dense intermediate layer, resulting in a more permeable structure.^61^

At elevated operating pressures, an increase in experimental standard deviations was identified, rendering the performance differences between the membranes with 1 and 5 wt % adsorbent loading less distinct. The increased system turbulence and pressure, combined with structural voids or defects induced by nanoparticle incorporation, likely account for these observed fluctuations.^59,60^

The permeation data for both pure water and the CIP solution across the PSf-20 series are summarized in Tables 5 and 6. Based on the flux profiles and the applied operating pressures, these membranes exhibit characteristic nanofiltration-like (NF-like) separation behavior. As expected, these membranes showed a reduction in flux when compared to the PSf-15 series. This is due to the increase in the viscosity of the solution and, therefore, a reduction in the speed of solvent exchange during the phase inversion process, resulting in a more compact and less permeable membrane.^50^

**Table 5:** Pure Water Flux Results for Membranes with 20 wt % PSf and Different Adsorbent Contents

	pure water flux (L m^–2^ h^–1^)		
membrane code	2 bar	4 bar	6 bar
M4	0.8 ± 0.1	1.3 ± 0.4	3.2 ± 1.4
M5	2.2 ± 1.2	2.8 ± 1.1	4.2 ± 2.2
M6	2.9 ± 1.7	3.6 ± 1.8	4.5 ± 1.9

**Table 6:** CIP Solution Flux Results for Membranes with 20 wt % PSf and Different Adsorbent Contents

	12.5 mg L^–1^ CIP solution flux (L m^–2^ h^–1^)		
membrane code	2 bar	4 bar	6 bar
M4	1.2 ± 0.2	2.1 ± 0.5	2.7 ± 2.1
M5	1.7 ± 0.8	3.5 ± 1.0	5.1 ± 3.7
M6	2.9 ± 1.7	5.5 ± 2.0	7.4 ± 5.0

Consistent with the PSf-15 series, the PSf-20 membranes modified with 1 and 5 wt % mSiO_2_ enhanced pure water flux at 2 bar compared to the reference. However, at higher operating pressures, this improvement became less distinct due to increased data variability, likely caused by structural defects intensified under these conditions. For the CIP solution, although the mean values suggest a general upward trend in permeate flux with higher nanoparticle loadings, the substantial overlap in standard deviations (Table 6) precludes a definitive statement of statistical superiority. This numerical behavior is qualitatively aligned with the enhanced hydrophilicity and a more interconnected porous structure featuring macrovoids observed in the characterization results.

Table 7 presents the CIP rejection values for all membranes synthesized. It was possible to identify a maximum rejection of 9.5 ± 3.5% for M1 at a pressure of 2 bar. With the addition of 1 and 5 wt % of SiO_2_, the rejection increased to 28.8 ± 6.9% and 48.3 ± 2.5%, respectively. This indicates that the incorporated mSiO_2_, in addition to promoting greater membrane permeate flux, enabled greater rejection, possibly due to the adsorbent action of the particle. It was also observed that the higher the operating pressure, the lower the rejection rate, a consequence of the greater flux for the same rejection action, which leads to a loss of efficiency in CIP removal.

**Table 7:** CIP Rejection Results for Membranes from 15 and 20 wt % PSf Solution and Different Adsorbent Contents

		rejection (%)		
membrane code	thickness (μm)	2 bar	4 bar	6 bar
M1	141 ± 14	9.50 ± 3.5	6.0 ± 2.8	5.9 ± 3.0
M2	138 ± 18	28.8 ± 6.9	25.1 ± 5.9	23.6 ± 5.2
M3	136 ± 14	48.3 ± 2.5	46.23 ± 3.2	45.6 ± 2.2
M4	135 ± 11	83.5 ± 12.0	79.9 ± 8.6	78.2 ± 8.3
M5	141 ± 16	83.3 ± 13.4	85.5 ± 6.4	77.3 ± 11.7
M6	140 ± 14	58.9 ± 4.7	56.0 ± 5.3	53.7 ± 6.7

Based on the literature and the characterization tests carried out for PSf −15 series membranes, the increase in CIP rejection may be related to several factors, such as the modification in the membrane structure, its hydrophilicity and its ability to adsorption. The results indicate that the incorporation of the SiO_2_ particle added CIP adsorption sites to the membrane capable of interacting with the drug molecules, reducing their quantity in the permeate.

For the PSf-20 series membranes, a higher CIP rejection rate was observed, even without the incorporation of nanoparticles, when compared to the PSf-15 series. However, the incorporation of 1 wt % of mSiO_2_ did not alter the nominal CIP rejection values compared to the reference membrane, reaching a maximum contaminant removal of 83.3 ± 13.4% in water.

Regarding the M6 membrane, a reduction in rejection capacity was observed, which can be attributed to an increase in the casting solution viscosity. This higher viscosity likely led to particle agglomeration during mixing or poor distribution within the membrane matrix, thereby hindering proper interaction with the adsorbate. Consequently, although the presence of mSiO_2_ enhanced the permeate flux, the impaired interaction between the CIP molecules and the adsorbent particles reduced the overall rejection performance. It is well established that poor compatibility between the inorganic filler and the polymer matrix compromises membrane rejection by inducing the formation of nonselective interfacial voids.^34^

Another factor that may influence the decline in CIP rejection is the increase in pore size induced by the incorporation of the adsorbent. The larger size of the pores, which is beneficial for water flux, tends to impair rejection, as they become larger and the exclusion of drug molecules by size becomes less efficient.

Based on the preliminary screening results obtained for the initial membrane series, an intermediate composition was subsequently developed as a targeted optimization step. This involved formulating an 18 wt % PSf casting solution, both without (M7) and with the incorporation of 1 wt % mSiO_2_ (M8). Tables 8 and 9 present the results obtained for these conditions.

**Table 8:** CIP Solution Flux Results for PSf-18 Series Membranes

	12.5 mg L^–1^ CIP solution flux (L m^–2^ h^–1^)		
membrane code	2 bar	4 bar	6 bar
M7	2.0 ± 1.0	2.9 ± 0.5	3.8 ± 1.6
M8	7.7 ± 1.0	12.0 ± 1.1	18.7 ± 2.1

**Table 9:** CIP Rejection Results for PSf-18 Series Membranes

		rejection (%)		
membrane code	thickness (μm)	2 bar	4 bar	6 bar
M7	135 ± 9	60.7 ± 3.0	53.9 ± 5.5	47.1 ± 4.0
M8	139 ± 12	73.6 ± 12.1	69.8 ± 8.5	62.0 ± 9.3

As shown in Table 8, PSf-18 series membranes exhibited an intermediate flux for the CIP solution between the PSf-15 and PSf-20 series. As identified for the PSf-15 series membranes, the incorporation of 1 wt % mSiO_2_ resulted in higher flux when compared to the reference membrane. These results indicate the structure incorporation reduces the resistance of the PSf membrane to pure water and CIP solution flux, likely due to the influence of the particles on the polymeric membrane morphology.

Increasing the PSf concentration from 15 to 18 wt % enhanced the baseline CIP rejection, as summarized in Table 9. Notably, the M8 membrane achieved rejection levels comparable to the denser PSf-20 series while maintaining a higher flux performance. These results further highlight the potential of mSiO_2_ modification to favorably optimize the trade-off between permeate flux and organic molecule rejection under low-pressure operation.

### 3.3 Proposed Mechanism for CIP Rejection in PSf/mSiO_2_ Membranes

To summarize these experimental findings, this section provides a proposed mechanism to elucidate the observed synergy between permeability and CIP rejection. The elucidation of the separation mechanism begins with the analysis of the contaminant’s chemical behavior. As demonstrated in Figure 11b, CIP exists predominantly in its zwitterionic form at pH 7. This state facilitates simultaneous interactions with the highly negative membrane surface, which exhibits a zeta potential of approximately −60 mV. While the pristine PSf membrane shows some intrinsic rejection through localized electrostatic repulsion and size exclusion, the incorporation of mSiO_2_ in the PSf matrix optimizes this system by expanding the available interaction area.^64^ The mSiO_2_ particles act as a potent pore-forming agent, accelerating the phase inversion kinetics to create a more interconnected macrostructure and the internal architecture appears to facilitate efficient contact between the solute and the pore walls.^69^ This suggests that the separation is not merely a surface phenomenon, but is deeply dependent on the internal transport pathways created during the membrane formation. Therefore, the enhanced separation efficiency is reasonably attributed to a synergistic mechanism that extends beyond simple size exclusion and surface interactions. To further evaluate the role of adsorption as a key driver of this performance, a global mass balance was performed on the M7 and M8 membranes to determine their dynamic and static adsorption capacities. The fundamental global mass balance equation to quantify the removal mechanism is expressed as follows

5 $$m_{total}=m_{permeate}+m_{\mathrm{retentate}}+m_{adsorbed}$$

where *m*_total_ is the initial mass of CIP in the feed solution, *m*_permeate_ is the CIP mass found in the permeate, *m*_retentate_ is the CIP mass remaining in the feed tank and *m*_adsorbed_ is the CIP mass adsorbed by the membrane. If the sum of the mass found in the permeate and the mass remaining in the feed tank is significantly lower than the initial mass, the deficit is attributed to the amount adsorbed within the membrane matrix.^70^

**Figure 11:**
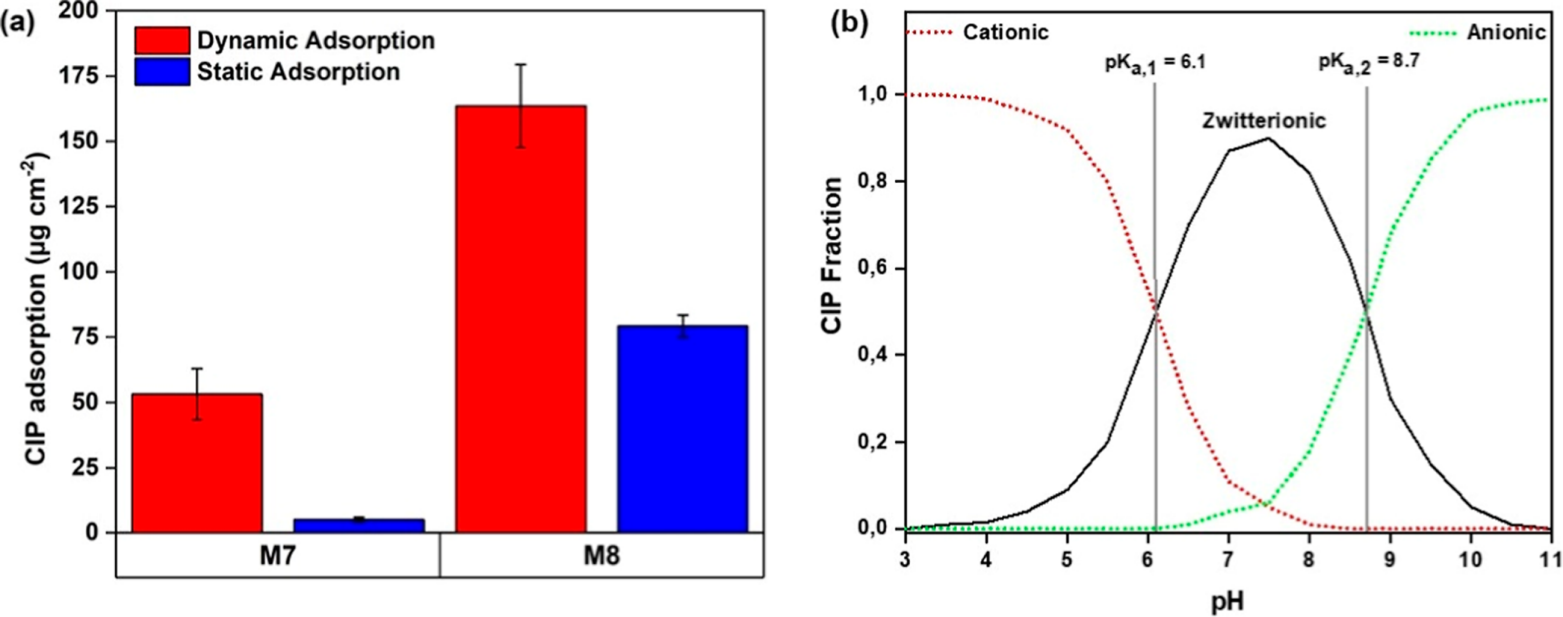
(a) Static and dynamic adsorption experiments of M7 and M8 membranes (b) CIP chemical speciation at various pH values.

For the static adsorption experiments, the M7 and M8 membranes samples (3 × 2 cm) were immersed in 50 mL of a CIP solution (12.5 mg L^–1^, pH 6.5) within a sealed vial. These vials were maintained at 25 °C for 24 h to ensure equilibrium was reached. The amount of adsorbed CIP was determined via UV–vis spectrophotometry. For both static and dynamic assays, the adsorbed mass was normalized by the membrane’s superficial area (cm^2^) to determine the area-specific adsorption capacities. The results are presented in Figure 11a.

Although the pristine membrane demonstrated an intrinsic ability to adsorb CIP, the results for the M8 membrane revealed a higher adsorption capacity. This behavior provides strong empirical evidence that the presence of mSiO_2_ intensifies the chemical affinity for the pharmaceutical, providing a high density of active sites that promote superior contaminant adsorption. Furthermore, as shown in Figure 11a, the area-normalized adsorbed mass for M8 reached approximately 163 μg cm^–2^ under dynamic conditions, an increase over static adsorption results. This indicates that the operating pressure helps mitigate diffusive resistance, forcing the zwitterionic contaminant into the active internal sites of the mSiO_2_ network. The enhanced CIP internal capture can be associated with the local hydrophilicity and negative charge characteristics of the mSiO_2_ nanoparticles, since EDS analysis confirmed their presence inside the matrix and pore walls. Consequently, as the pressurized feed forces the zwitterionic CIP into the pore structure, the local availability of hydrophilic silanol groups inside the channels creates a highly favorable wetting environment.^60^ At near-neutral operating pH, the deprotonation of these internal silanol groups generates a localized negative charge density within the confinement of the mesoporous network. This specific environment promotes targeted electrostatic or dipole–dipole interactions with the polar/zwitterionic structure of CIP.^32^ Therefore, the convective flow not only overcomes diffusive resistance but actively drives the contaminant into intimate contact with these internal active sites, maximizing this local adsorptive synergy. The mSiO_2_ mesopores appear to act as molecular sites capturing the solute much more efficiently than the flat polymer surface, supporting the development of a high-permeability adsorptive membrane composite.^50^

The performance of the synthesized membranes is governed by complex molecular interactions between the PSf matrix, mSiO_2_ and CIP. By correlating structural and rheological insights with the pore architecture and surface properties, it is possible to propose a comprehensive transport and separation mechanism that aligns with both static and dynamic adsorption results.^26^ The mass balance calculations strongly support the proposed synergy between Donnan exclusion and adsorption capacity. Given that the average membrane pore radius significantly exceeds the molecular dimensions of the CIP molecule 1.5 nm, retention is likely governed by a combination of Donnan exclusion and convective-enhanced adsorption within the mSiO_2_ micro and mesopores. This multifaceted approach clarifies how the incorporation of nanoparticles allows the membrane to substantially improve the traditional performance trade-off, functioning as an integrated adsorptive nanocomposite.^15^

The results obtained for these membranes are highly promising when compared to other matrix configurations reported in the literature for antibiotic removal (Table 10). It is worth noting that this literature comparison is primarily qualitative, serving to contextualize the performance of the developed nanocomposites within the current research landscape. Because parameters such as feed concentration, pH, and solution matrix vary widely among the reported studies, Table 10 does not constitute a strict benchmarking tool, but rather highlights the competitive potential of the present membrane. Although some studies report slightly higher rejection values, the membrane engineered in this work delivers a highly favorable balance of CIP removal under a remarkably low operating pressure, while maintaining a pure water flux consistent with the NF-like process.

**Table 10:** Comparison of the Results Obtained in the Present Work with Other Studies in the Literature

membrane composition	antibiotic	pressure (bar)	maximum rejection (%)
PSf/TPU/UiO-66 (NH_2_)^71^	tetracycline	5.00	99.40
PSf/M-SiO_2_ (this study)	CIP	2.00	83.3 ± 13.40
PSf/polyacrylate acid^72^	amoxicillin	3.00	91.00
PSf/SiO_2_ ^13^	amoxicillin	5.00	89.80
PSf/diatomite^73^	neomycin	1.38	85.90
PSf/PEG^37^	amoxicillin	3.00	85.00

While these high separation and permeation efficiencies demonstrate the significant potential of mSiO_2_/PSf MMMs, it is essential to note that these results were obtained using a model CIP solution under controlled laboratory conditions. The practical relevance of this membrane configuration must be further validated through comprehensive assessments of complex operational challenges. Specifically, future studies are required to evaluate performance in real wastewater matrices, as well as to conduct thorough investigations into membrane fouling, long-term operational stability, and reusability cycles. These investigations are essential future steps that will build upon our current findings, ultimately enabling the implementation of this robust membrane configuration in sustainable, large-scale water treatment technologies.

## 4 Conclusion

Membranes exhibiting varying PSf concentrations, with and without the incorporation of 1 and 5 wt % mSiO_2_ structures, were fabricated, characterized, and evaluated for permeation and CIP rejection performance under controlled laboratory conditions. The hydrophilic and porous nature of mSiO_2_ significantly influenced membrane performance. SEM analysis indicated that the incorporation altered the solvent–nonsolvent exchange rate during phase inversion, inducing structural and morphological changes. The incorporation of mSiO_2_ enhanced permeate flux, particularly in membranes with lower polymer concentrations, while maintaining or improving antibiotic rejection depending on the tested conditions. At 2 bar, the M8 membrane achieved a rejection rate (73.6 ± 12.1%) comparable to that of the M5 membrane, while exhibiting a 4-fold increase in flux under the same operating conditions. The presence of mSiO_2_ provides internal active sites that act as sites for CIP molecules, favorably optimizing the balance between flux and rejection through an adsorption–membrane synergism. These findings strongly suggest that the Donnan effect and adsorption mechanisms play a predominant role in governing the high rejection performance of the hybrid matrices. The synergy between PSf membrane filtration properties, CIP adsorption capacity, and the hydrophilic nature of mSiO_2_ nanoparticles resulted in a high-performance nanocomposite membrane, offering valuable insights for the development of energy-efficient membranes for pharmaceutical contaminant removal.

## Data Availability

The data underlying this study are not publicly available due to intellectual property protection. The data are available from the corresponding author upon reasonable request, pending institutional approval and subject to a formal data transfer and nondisclosure agreement.

## References

[1] Vaudin P., Augé C., Just N., Mhaouty-Kodja S., Mortaud S., Pillon D. (2022). When pharmaceutical
drugs become
environmental pollutants: Potential neural effects and underlying
mechanisms. Environ. Res..

[2] Gandomkarzadeh M., Moghimi H., Mahboubi A. (2020). Evaluation of the Effect of Ciprofloxacin
and Vancomycin on Mechanical Properties of PMMA Cement; a Preliminary
Study on Molecular Weight. Sci. Rep..

[3] Sharma P., Jain A., Jain S., Pahwa R., Yar M. (2010). Ciprofloxacin:
review on developments in synthetic, analytical, and medicinal aspects. J. Enzyme Inhib. Med. Chem..

[4] Couto C. F., Lange L. C., Amaral M. C. S. (2018). A critical review
on membrane separation
processes applied to remove pharmaceutically active compounds from
water and wastewater. J. Water Process Eng..

[5] Singh R., Singh A., Kumar S., Giri B., Kim K. (2019). Antibiotic
resistance in major rivers in the world: A systematic review on occurrence,
emergence, and management strategies. J. Clean.
Prod..

[6] Lu Z., Ma Y., Zhang J., Fan N., Huang B., Jin R. (2020). A critical
review of antibiotic removal strategies: Performance and mechanisms. J. Water Process Eng..

[7] Phoon B. L., Ong C. C., Saheed M. S. M., Show P. L., Chang J.-S., Ling T. C., Lam S. S., Juan J. C. (2020). Conventional and
Emerging Technologies for Removal of Antibiotics from Wastewater. J. Hazard. Mater..

[8] Yan Y., Pengmao Y., Xu X., Zhang L., Wang G., Jin Q., Chen L. (2020). Migration
of antibiotic ciprofloxacin during phytoremediation
of contaminated water and identification of transformation products. Aquat. Toxicol..

[9] Puri N., Gupta A., Mishra A. (2021). Recent advances
on nano-adsorbents
and nanomembranes for the remediation of water. J. Clean. Prod..

[10] Al-Buriahi A. K., Al-shaibani M., Mohamed R., Al-Gheethi A. A., Sharma A., Ismail N. (2022). Ciprofloxacin
removal from non-clinical
environment: A critical review of current methods and future trend
prospects. J. Water Process Eng..

[11] Dolar D., Vuković A., Ašperger D., Košutić K. (2011). Effect of
water matrices on removal of veterinary pharmaceuticals by nanofiltration
and reverse osmosis membrane. J. Environ. Sci..

[12] Taheran M., Brar S. K., Verma M., Surampalli R. Y., Zhang T. C., Valero J. R. (2016). Membrane processes
for removal of
pharmaceutically active compounds (PhACs) from water and wastewaters. Sci. Total Environ..

[13] Shakak M., Rezaee R., Maleki A., Jafari A., Safari M., Shahmoradi B., Daraei H., Lee S. (2020). Synthesis and characterization
of nanocomposite ultrafiltration membrane (PSF/PVP/SiO_2_) and performance evaluation for the removal of amoxicillin from
aqueous solutions. Environ. Technol. Innov..

[14] Zyłła R., Foszpańczyk M., Kamínska I., Kudzin M., Balcerzak J., Ledakowicz S. (2022). Impact of
Polymer Membrane Properties on the Removal
of Pharmaceuticals. Membranes.

[15] Cevallos-Mendoza J., Alay-Macias G., Figueira F., Araujo A., Amorim C., Rodríguez-Díaz J. M., Montenegro M. (2024). Nanocomposite
HKUST-1@polysulfone membrane for the adsorptive removal of tetracyclines
in waters. Sep. Purif. Technol..

[16] Dharupaneedi S. P., Nataraj S. K., Nadagouda M. N., Reddy K. R., Shukla S. S., Aminabhavi T. M. (2019). Membrane-based
separation of potential emerging pollutants. Sep. Purif. Technol..

[17] Urtiaga A. (2021). Electrochemical
technologies combined with membrane filtration. Curr. Opin. Electrochem..

[18] Chadha U., Selvaraj S. K., Thanu S., Cholapadath V., Mathew Abraham A., Zaiyan M., Manoharan M., Paramsivam V. (2022). A review of the function of using carbon nanomaterials
in membrane filtration for contaminant removal from wastewater. Mater. Res. Express.

[19] Koyuncu I., Arikan O. A., Wiesner M. R., Rice C. (2008). Removal of hormones
and antibiotics by nanofiltration membranes. J. Membr. Sci..

[20] Serbanescu O., Voicu S. I., Thakur V. K. (2020). Polysulfone
functionalized membranes:
Properties and challenges. Mater. Today Chem..

[21] Ramachandran S., Sathishkumar P. (2023). Membrane-based techniques for pollutants
removal: An
outlook on recent advancements. Curr. Opin.
Environ. Sci. Health.

[22] Lu Z., Yang Y., Liu Z., Wen H., Xu J., Chen J. P. (2025). Boehmite nanoparticles and poly­(vinyl
alcohol) modified
nanofiltration membrane for efficient treatment of antibiotics and
salt containing aqueous solutions: Design and evaluation. Sep. Purif. Technol..

[23] Hu Z., Yin Z., Chen Y., Wen T., Li F., Yang W. (2024). Development
of conductive two-dimensional metal-organic frameworks self-cleaning
membrane for enhanced antibiotics rejection and sustainable fouling
mitigation. J. Membr. Sci..

[24] Yang Z., Lin Q., Zeng G., Zhao S., Yan G., Ang M. B. M. Y., Chiao Y., Pu S. (2023). Ternary hetero-structured BiOBr/Bi2MoO6@MXene
composite membrane: Construction and enhanced removal of antibiotics
and dyes from water. J. Membr. Sci..

[25] Arefi-Oskoui S., Khataee A., Behrouz S. J., Vatanpour V., Gharamaleki S., Orooji Y., Safarpour M. (2022). Development
of MoS2/O-MWCNTs/PES blended membrane for efficient removal of dyes,
antibiotic, and protein. Sep. Purif. Technol..

[26] Al-Timimi D. A. H., Alsalhy Q. F., AbdulRazak A. A., Drioli E. (2022). Novel polyether sulfone/polyethylenimine
grafted nano-SiO_2_ nanocomposite membranes: Interaction
mechanism and ultrafiltration performance. J.
Membr. Sci..

[27] Nikfarjam Z., Heravi M., Bozorgmehr M. (2022). Removal of
Cefotaxime Antibiotics
from Hospital Wastewater using TiO_2_ and SiO_2_ Nanoparticles-Modified Polyethersulfone Membrane: Experimental and
Computational Study. Res. Sq..

[28] Sargolzaei Z., Chianeh F. N., Shamsodin M. (2023). Improvement
of characterization and
application of Polyethersulfone membrane by CeO_2_@SiO_2_ nanoparticles for drug pollutant removal. J. Porous Mater..

[29] Waheed A., Baig U., Jillani S. (2023). Optimization
of amine functionalization
of MCM-41 for its covalent decoration in nanofiltration membranes
for purification of saline- and micropollutant-contaminated feeds. Environ. Sci.:Water Res. Technol..

[30] Jillani S., Waheed A., Baig U., Aljundi I. (2024). Fabrication of highly
efficient nanofiltration membranes decorated with praseodymium based
triamino functionalized MCM-41 for desalination and micropollutants
removal. Arabian J. Chem..

[31] Sharma J., Polizos G. (2020). Hollow Silica Particles: Recent Progress and Future
Perspectives. Nanomaterials.

[32] Assis D., Santos E., Eiras D. (2024). Efficient and reusable mesoporous
silica structures for ciprofloxacin removal from water media. J. Water Process Eng..

[33] Mulyati S., Muchtar S., Yusuf M., Arahman N., Sofyana S., Rosnelly C., Fathanah U., Takagi R., Matsuyama H., Shamsuddin N., Bilad M. R. (2020). Production of High Flux Poly­(ether
sulfone) Membrane Using Silica Additive Extracted from Natural Resource. Membranes.

[34] Wu H., Tang B., Wu P. (2014). Development
of novel SiO_2_–GO nanohybrid/polysulfone membrane
with enhanced performance. J. Membr. Sci..

[35] Teli S., Molina S., Sotto A., Calvo E. G., Abajo J. d. (2013). Fouling
Resistant Polysulfone–PANI/TiO_2_ Ultrafiltration
Nanocomposite Membranes. Ind. Eng. Chem. Res..

[36] Ahmadipouya S., Mousavi S. A., Shokrgozar A., Mousavi D. V. (2022). Improving dye removal
and antifouling performance of polysulfone nanofiltration membranes
by incorporation of UiO-66 metal-organic framework. J. Environ. Chem. Eng..

[37] Derakhsheshpoor R., Homayoonfal M., Mehrnia M. (2013). Amoxicillin separation from pharmaceutical
wastewater by high permeability polysulfone nanofiltration membrane. J. Environ. Health Sci. Eng..

[38] Ajayi S. M., Olusanya S. O., Abimbade S. F., Olumayede E. G., Faboya O. L., Malomo D., Akintayo C. O. (2025). Synthesis
of Mesoporous
Silica Nanoparticles from Oil Palm Leaves: Mechanism and Characterization
Using Optical Microscope. Next. Res..

[39] Sing K. S. W., Everett D. H., Haul R. A. W., Moscou L., Pierotti R. A., Rouquerol J., Siemieniewska T. (1985). Reporting
Physisorption Data for
Gas/Solid Systems with Special Reference to the Determination of Surface
Area and Porosity (Recommendations 1984). Pure
Appl. Chem..

[40] Thommes M., Kaneko K., Neimark A. V., Olivier J. P., Rodriguez-Reinoso F., Rouquerol J., Sing K. S. W. (2015). Physisorption of Gases, with Special
Reference to the Evaluation of Surface Area and Pore Size Distribution
(IUPAC Technical Report). Pure Appl. Chem..

[41] Mousavi S., Raveshiyan S., Amini Y., Zadhoush A. (2023). A critical
review with
emphasis on the rheological behavior and properties of polymer solutions
and their role in membrane formation, morphology, and performance. Adv. Colloid Interface Sci..

[42] Omidvar M., Soltanieh M., Mousavi S., Saljoughi E., Moarefian A., Saffaran H. (2015). Preparation of hydrophilic nanofiltration
membranes for removal of pharmaceuticals from water. J. Environ. Health Sci. Eng..

[43] Shukla A., Alam J., Alhoshan M. (2025). Rheological
behavior of polyphenylsulfone-organic
additives solutions: a key factor in membrane fabrication process. Polym.-Plast. Technol. Mater..

[44] Yang Y., Zhang H., Wang P., Zheng Q., Li J. (2007). The influence
of nano-sized TiO_2_ fillers on the morphologies and properties
of PSF UF membrane. J. Membr. Sci..

[45] Salahshoori I., Nasirian D., Rashidi N., Hossain M., Hatami A., Hassanzadeganroudsari M. (2021). The effect
of silica nanoparticles
on polysulfone–polyethylene glycol (PSF/PEG) composite membrane
on gas separation and rheological properties of nanocomposites. J. Polym. Sci. (N. Y. NY U. S.).

[46] Muntha S., Ajmal M., Naeem H., Kausar A., Zia M., Siddiq M. (2019). Synthesis, Properties, and Applications of Polysulfone/Polyimide
Nanocomposite Membrane Reinforced with Silica Nanoparticles. Polym. Compos..

[47] Mohamed F., Hameed T., Turk G. (2021). Structure–dynamic
properties
relationships in poly­(ethylene oxide)/silicon dioxide nanocomposites:
dielectric relaxation study. J. Polym. Sci.
(N. Y. NY U. S.).

[48] Lufrano F., Squadrito G., Patti A., Passalacqua E. (2000). Sulfonated
Polysulfone as Promising Membranes for Polymer Electrolyte Fuel Cells. J. Appl. Polym. Sci..

[49] Singh K., Devi S., Bajaj H., Ingole P., Choudhari J., Bhrambhatt H. (2014). Optical resolution
of racemic mixtures of amino acids
through nanofiltration membrane process. Sep.
Sci. Technol..

[50] Alharbi A., Desouky A., Bayoumi S., Shahat A., Ali M. (2022). Hybrid polysulfone/mesoporous
silica ultrafiltration membranes for industrial wastewater treatment. Desalin. Water Treat..

[51] Muhamad M., Salim M., Lau W., Yuzir M., Yunus S. (2015). Fabrication
of mixed matrix membrane incorporated with modified silica nanoparticles
for bisphenol A removal. J. Teknol..

[52] Yin J., Kim E., Yang J., Deng B. (2012). Fabrication of a novel thin-film
nanocomposite (TFN) membrane containing MCM-41 silica nanoparticles
(NPs) for water purification. J. Membr. Sci..

[53] Ananth A., Arthanareeswaran G., Mok Y. (2014). Effects of in situ and ex situ formations
of silica nanoparticles on polyethersulfone membrane. J. Polym. Sci. (N. Y. NY U. S.).

[54] Song H., Kim C. (2013). Fabrication and properties of ultrafiltration
membranes composed
of polysulfone and poly­(1-vinylpyrrolidone) grafted silica nanoparticles. J. Membr. Sci..

[55] Jamalludin M. R., Harun Z., Hubadillah S. K., Basri H., Ismail A. F., Othman M. H. D., Shohur M., Yunos M. Z. (2016). Antifouling polysulfone
membranes blended with green SiO_2_ from rice husk ash (RHA)
for humic acid separation. Chem. Eng. Res. Des..

[56] Rodrigues R., Mierzwa J. C., Vecitis C. D. (2019). Mixed matrix
polysulfone/clay nanoparticles
ultrafiltration membranes for water treatment. J. Water Process Eng..

[57] Zahedi S. M., Karimi M., de Silva J. A. T. (2020). The
use of nanotechnology to increase
quality and yield of fruit crops. J. Sci. Food
Agric..

[58] Zhang Z., Song Y., Xie L., Liu Q., Li J., Zhang B. (2024). A loose polyethersulfone hybrid nanofiltration
membrane incorporated
with polyethyleneimine-decorated silica nanoparticles for highly-efficient
dye/salt separation. Chem. Eng. J..

[59] Mamah S. C., Goh P. S., Ng B. C., Abdullah M. S., Ismail A. F., Samavati A., Ahmad N., Raji Y. O. (2024). The utilization
of chitin and chitosan as green modifiers in nanocomposite membrane
for water treatment. J. Water Process Eng..

[60] Huang J., Zhang K., Wang K., Xie Z., Ladewig B. P., Wang H. (2012). Fabrication of polyethersulfone-mesoporous
silica nanocomposite ultrafiltration
membranes with antifouling properties. J. Membr.
Sci..

[61] Saberi S., Shamsabadi A. A., Shahrooz M., Sadeghi M., Soroush M. (2018). Improving
the Transport and Antifouling Properties of Poly­(vinyl chloride) Hollow-Fiber
Ultrafiltration Membranes by Incorporating Silica Nanoparticles. ACS Omega.

[62] Ahn J., Chung W. J., Pinnau I., Guiver M. D. (2008). Polysulfone/silica
nanoparticle mixed-matrix membranes for gas separation. J. Membr. Sci..

[63] Jadav G. L., Singh P. S. (2009). Synthesis of novel
silica-polyamide nanocomposite membrane
with enhanced properties. J. Membr. Sci..

[64] Dong L., Huang X., Wang Z., Yang Z., Wang X., Tang C. Y. (2016). A thin-film nanocomposite
nanofiltration membrane prepared
on a support with in situ embedded zeolite nanoparticles. Sep. Purif. Technol..

[65] Ghosh A., Bindal R. C., Tewari P. K. (2015). Preparation
of Silica-Polysulfone
Based High Flux Fouling Resistant Nanocomposite Ultrafiltration Membranes
for Separation of Proteins, Polysaccharides and Humic Substances. J. Macromol. Sci., Part A.

[66] Bidsorkhi H. C., Riazi H., Emadzadeh D., Ghanbari M., Matsuura T., Lau W. J., Ismail A. F. (2016). Preparation
and characterization
of a novel highly hydrophilic and antifouling polysulfone/nanoporous
TiO_2_ nanocomposite membrane. Nanotechnology.

[67] Hoek E. M. V., Agarwal G. K. (2006). Extended DLVO interactions
between spherical particles
and rough surfaces. J. Colloid Interface Sci..

[68] Mohammad A. W., Teow Y. H., Ang W. L., Chung Y. T., Oatley-Radcliffe D. L., Hilal N. (2015). Nanofiltration membranes
review: Recent advances and future prospects. Desalination.

[69] Sen D., Ghosh A. K., Majumder S., Bindal R. C., Tiwari P. K. (2014). Novel polysulfone-spray-dried
silica composite membrane for water purification: Preparation, characterization
and performance evaluation. Sep. Purif. Technol..

[70] Mulder, M. Basic Principles of Membrane Technology, 2 ed.; Kluwer Academic Publishers: Dordrecht, 1996.

[71] Guo J., Huang M., Gao P., Zhang Y., Chen H., Zheng S., Mu T., Luo X. (2020). Simultaneous robust
removal of tetracycline and tetracycline resistance genes by a novel
UiO/TPU/PSF forward osmosis membrane. Chem.
Eng. J..

[72] Homayoonfal M., Mehrnia M. (2014). Amoxicillin separation
from pharmaceutical solution
by pH sensitive nanofiltration membranes. Sep.
Purif. Technol..

[73] Paidi M., Polisetti V., Damarla K., Singh P. S., Mandal S., Ray P. (2022). 3D Natural
Mesoporous Biosilica-Embedded Polysulfone Made Ultrafiltration
Membranes for Application in Separation Technology. Polym..

